# The InBIO Barcoding Initiative Database: contribution to the knowledge on DNA barcodes of cuckoo wasps, with the description of new species from the Iberian Peninsula (Hymenoptera, Chrysididae)

**DOI:** 10.3897/BDJ.11.e98743

**Published:** 2023-03-01

**Authors:** Paolo Rosa, Thomas Wood, Teresa Luísa L. Silva, Joana Veríssimo, Vanessa A. Mata, Denis Michez, Pedro Beja, Sónia Ferreira

**Affiliations:** 1 University of Mons, Research Institute for Biosciences, Laboratory of Zoology, Place du parc 20, 7000, Mons, Belgium University of Mons, Research Institute for Biosciences, Laboratory of Zoology, Place du parc 20, 7000 Mons Belgium; 2 CIBIO, Centro de Investigação em Biodiversidade e Recursos Genéticos, InBIO Laboratório Associado, Campus de Vairão, Universidade do Porto, 4485-661 Vairão, Vila do Conde, Portugal CIBIO, Centro de Investigação em Biodiversidade e Recursos Genéticos, InBIO Laboratório Associado, Campus de Vairão, Universidade do Porto, 4485-661 Vairão Vila do Conde Portugal; 3 BIOPOLIS Program in Genomics, Biodiversity and Land Planning, CIBIO, Campus de Vairão, 4485-661 Vairão, Vila do Conde, Portugal BIOPOLIS Program in Genomics, Biodiversity and Land Planning, CIBIO, Campus de Vairão, 4485-661 Vairão Vila do Conde Portugal; 4 CIBIO, Centro de Investigação em Biodiversidade e Recursos Genéticos, InBIO Laboratório Associado, Instituto Superior de Agronomia, Universidade de Lisboa, Lisboa, Portugal CIBIO, Centro de Investigação em Biodiversidade e Recursos Genéticos, InBIO Laboratório Associado, Instituto Superior de Agronomia, Universidade de Lisboa Lisboa Portugal

**Keywords:** Portugal, Spain, Italy, DNA barcode, mitochondrial DNA, Cytochrome c oxidase subunit I (COI)

## Abstract

**Background:**

DNA barcoding technologies have provided a powerful tool for the fields of ecology and systematics. Here, we present a part of the InBIO Barcoding Initiative Database: contribution to the knowledge on DNA barcodes of cuckoo wasps (Hymenoptera, Chrysididae) dataset representing 144 specimens and 103 species, covering approximately 44% of the Iberian and 21% of the European fauna. The InBIO Barcoding Initiative (IBI – DNA Barcoding Portuguese terrestrial invertebrate biodiversity) aims to fill the barcoding gap for the terrestrial invertebrate taxa. All DNA extractions are deposited in the IBI collection at CIBIO, Research Center in Biodiversity and Genetic Resources and specimens are deposited in the University of Mons collection (Belgium) and in the Natur-Museum in Lucerne (Switzerland).

**New information:**

This dataset increases the knowledge on the DNA barcodes and distribution of 102 species of cuckoo wasps. A total of 52 species, from 11 different genera, were new additions to the Barcode of Life Data System (BOLD), with DNA barcodes for another 44 species added from under-represented taxa in BOLD. All specimens have their DNA barcodes publicly accessible through the BOLD online database. Nine cuckoo wasp species are newly recorded for Portugal. Additionally, two new species for science are described: *Chrysiscrossi* Rosa, sp. nov. from southern Portugal and *Hedychridiumcalcarium* Rosa, sp. nov. from eastern Spain. Several taxonomic changes are proposed and *Hedychrumrutilans* Dahlbom, 1845 is found to consist of two different taxa that can be found in sympatry, *Hedychrumrutilans* s. str. and *Hedychrumviridaureum* Tournier, 1877 stat. nov. *Stilbumwestermanni* Dahlbom, 1845 stat. nov. is confirmed as distinct from *Stilbumcalens* (Fabricius, 1781), with the latter species not confirmed as present in Iberia; barcoded *Stilbum* material from Australia is distinct and represents *Stilbumamethystium* (Fabricius, 1775) sp. resurr.; Portuguese material identified as *Hedychridiumchloropygum* Buysson, 1888 actually belongs to *Hedychridiumcaputaureum* Trautmann & Trautmann, 1919, the first confirmed record of this species from Iberia. *Philoctetesparvulus* (Dahlbom, 1845) is confirmed to be a synonym of *Philoctetespunctulatus* (Dahlbom, 1845). *Chrysislusitanica* Bischoff, 1910 is confirmed as a valid species. *Chrysishebraeica* Linsenmaier, 1959 stat. nov. is raised to species status.

## Introduction

In Europe, the diversity of cuckoo wasps is highest in the Mediterranean region, with relatively few species found in the north (Paukkunen et al. 2014, Paukkunen et al. 2015) and the British Isles (Morgan 1984). Chrysidids are more common in southern European countries in part due to their ecology, since most species are heliophilous and thermophilous, favouring warm and sunny habitats. Another reason is their reproductive biology, as the number of host species of bees and aculeate wasps is also greater in Mediterranean countries (Michez et al. 2019).

The total number of valid cuckoo wasp species is approximately 2,800 (Rosa 2017). Of this world total, about 480 have been recorded from Europe, plus 135 accepted subspecies, whose possible specific rank has yet to be evaluated (Mitroiu et al. 2015). In Portugal, a total of 130 species and four subspecies are known to date, but this number is likely to be far from the true total given the much larger number of species reported from neighbouring Spain (e.g. González et al. 1999, González et al. 2009, Mingo and Gayubo 1981, Mingo and Gayubo 1986). Mingo (1994) compiled the most recent faunistic survey for the Iberian Peninsula, including identification keys. In this important monograph, Portuguese species are not clearly separated from Spanish ones. This volume is a valuable guide for beginners, yet includes only 170 species for the Iberian Peninsula, overlooking many of the species described or published from Spain. The real number of species exceeds 230 known taxa. However, new field research on Portuguese bees and aculeate wasps (e.g. Rosa et al. 2015, Rosa et al. 2015, Rosa and Vårdal 2015, Rosa and Xu 2015, Baldock et al. 2018, Baldock et al. 2020, Cross et al. 2021) has reinvigorated work on the Iberian cuckoo wasp fauna and a new illustrated catalogue of the Portuguese fauna is in preparation, including new records for the country and for Europe.

Despite the fact that the Iberian Chrysididae fauna is one of the richest in Europe (Mingo 1994), this fauna has essentially never been investigated using molecular tools, with only a handful of DNA barcodes sequences available from a small selection of species (e.g. Pauli et al. (2019)). To a certain extent, this is a function of the limited number of studies that have presented barcode data for West Palaearctic Chrysididae in general (Niehuis and Wägele 2004, Soon and Saarma 2011, Soon et al. 2014, Paukkunen et al. 2015, Orlovskyté et al. 2016, Roslin et al. 2021). The present work represents the first attempt to generate DNA barcodes for Iberian Chrysididae at a faunal level and, thus, represents a major step in documenting the genetic diversity in the Mediterranean cuckoo wasp fauna.

## Materials and methods

**Figure 1.**
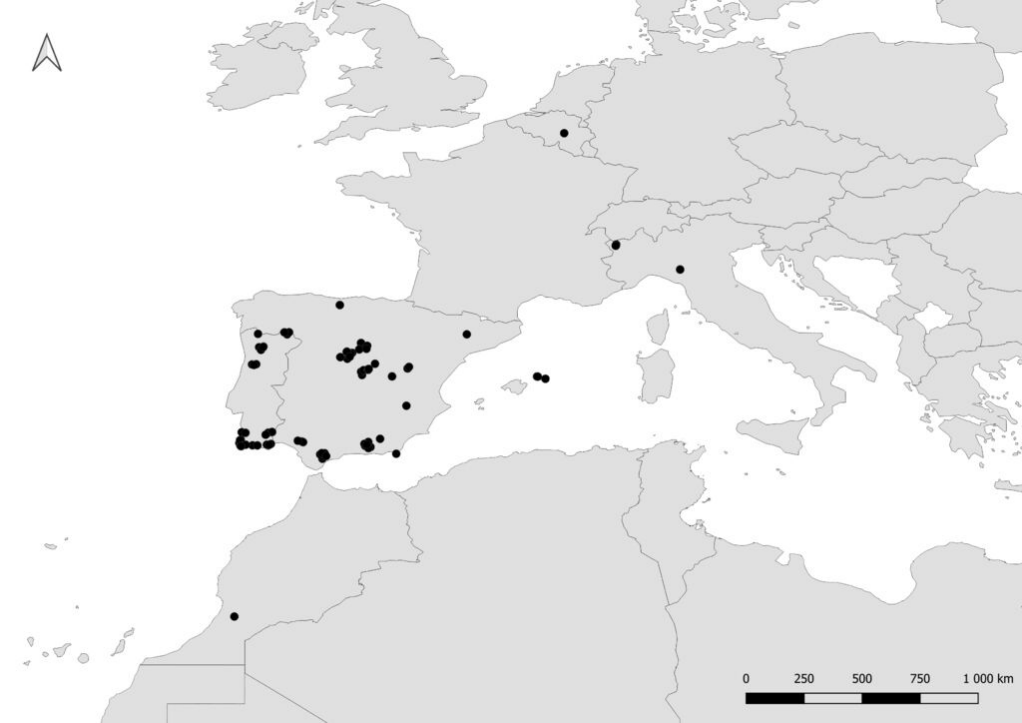
Map of the localities where cuckoo wasps samples were collected.

**Figure 2.**
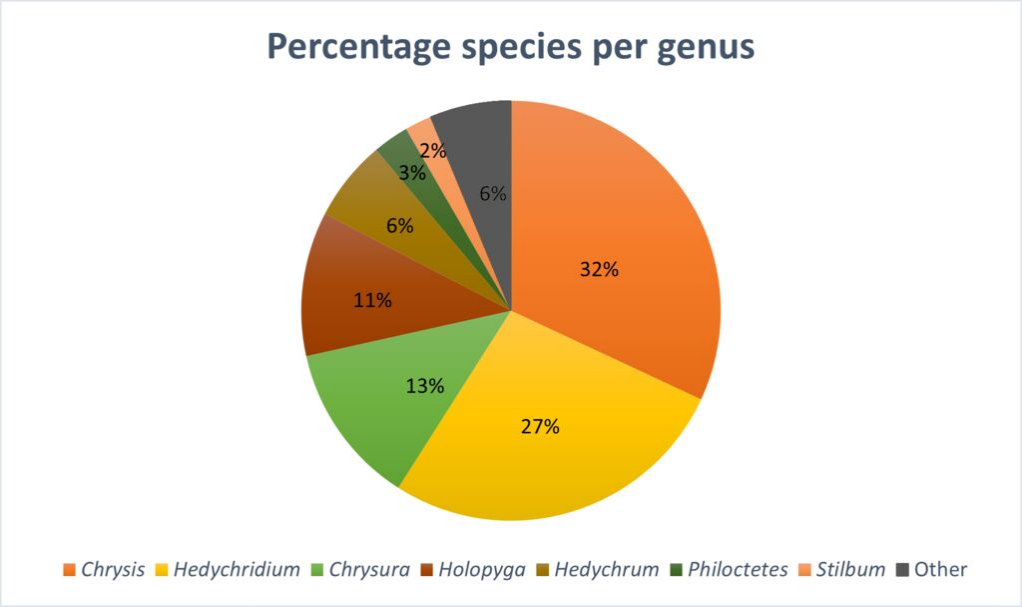
Distribution of species (%) for each Chrysididae genus present in the dataset. Genera represented by less than 2% of species were lumped together.

This dataset is composed of data relating to 144 Chrysididae specimens. Specimens were collected during field expeditions in the Iberian Peninsula, Belgium, Italy and Morocco from 2014 to 2022 by T.J. Wood, I. Cross (Dorchester, UK) and P. Rosa (Fig. 1, Table 1). Specimens were pinned and dried and are preserved in the collection of T.J. Wood at the University of Mons (Belgium); Italian specimens are preserved in ethanol (98%) and preserved in the collection of P. Rosa at the University of Mons (Belgium). Holotypes of the newly-described species are deposited in the Natur-Museum (Lucerne, Switzerland – **NMLU**) and paratypes are deposited in the following private collections: **PRC** (Paolo Rosa Collection, Bernareggio, Italy), **TWC** (Thomas J. Wood Collection, Mons, Belgium); **ICC** (Ian Cross Collection, Briantspuddle, Dorset, United Kingdom). The majority of specimens were determined to species level, though some specimens in challenging or unclear taxonomic groups were identified as 'cf.' or simply to the species group. Overall, 103 species are represented in the dataset. These species belong to 13 genera (Fig. 2).

Specimens were captured with an entomological net, euthanised by exposure to ethyl acetate and pinned and dried within 24 hours to achieve maximum suitability for DNA extraction and amplification.

DNA was extracted using a QIAmp DNA Micro Kit that is designed to extract higher concentrations of genetic material from samples with small amounts of DNA. DNA amplification was performed using two different primer pairs, that amplify partially overlapping fragments (LC + BH) of the 658 bp barcoding region of the COI mitochondrial gene (Folmer et al. 1994). We used the primers FwhF1 (Vamos et al. 2017) + C_R (Shokralla et al. 2015) for LC and BF3 (Elbrecht et al. 2019) + BR2 (Elbrecht and Leese 2017) for BH amplification, all modified with Illumina adaptors. PCRs were performed in 10 µl reactions, containing 5 µl of Multiplex PCR Master Mix (Qiagen, Germany), 0.3 µl of each 10 mM primer and 1-2 µl of DNA, with the remaining volume in water. PCR cycling conditions consisted in an initial denaturation at 95ºC for 15 min, followed by 45 cycles of denaturation at 95ºC for 30 sec, annealing at 45ºC for 45 sec and extension at 72ºC for 45 sec, with a final elongation step at 60ºC for 10 min. All DNA extracts were deposited in the IBI collection.

Successful amplification was validated through 2% agarose gel electrophoresis and samples selected for sequencing followed for a second PCR, where Illumina P5 and P7 adapters with custom 7 bp long barcodes were attached to each PCR product. The index PCR was performed in a volume of 10 µl, including 5 µl of KAPA HiFi PCR Kit (KAPA Biosystems, U.S.A.), 0.5 µl of each 10 mM indexing primer and 2 µl of diluted PCR product (usually 1:4). PCR cycling conditions were as before, except that only 10 cycles were performed and at an annealing temperature of 55ºC. The amplicons were purified using AMPure XP beads (New England Biolabs, U.S.A.) and quantified using NanoDrop 1000 (Thermo Scientific, U.S.A.). Clean PCR products were then pooled equimolarly per fragment. Each pool was quantified with KAPA Library Quantification Kit Illumina® Platforms (KAPA Biosystems, U.S.A.) and the 2200 Tapestation System (Agilent Technologies, California, USA) was used for fragment length analysis prior to sequencing (Paupério et al. 2018). DNA sequencing was done at CIBIO facilities on an Illumina MiSeq benchtop system, using a V2 MiSeq sequencing kit (2x 250 bp).

Illumina sequencing reads were processed using OBITools (Boyer et al. 2016) and VSEARCH (Rognes et al. 2016). Briefly, paired-end reads were aligned, collapsed into exact sequence variants, filtered by length, denoised and checked for chimeras. The resulting sequences from both LC and BH fragments of each sample were further assembled using CAP3 (Huang and Madan 1999) to produce a single 658 bp contig per sample.

All sequences in the dataset were submitted to BOLD and GenBank databases and, to each sequenced specimen, the morphological identification was contrasted with the results of the BLAST of the newly-generated DNA barcodes in the BOLDIdentification Engine. In order to clarify the taxonomic status of problematic groups, DNA barcodes generated here were analysed with other sequences from across the West Palaearctic downloaded from BOLD and GenBank. Sequences (658 bp) were aligned using SeaView (Gouy et al. 2010) and a neighbour-joining phylogeny was run with 10,000 bootstraps. Intra- and interspecific distances were calculated using MEGA-X (Kumar et al. 2018).

### Acronyms and abbreviations

**NMLU** = Natur-Museum, Lucerne, Switzerland

**PRC** = Paolo Rosa Collection, Bernareggio, Italy

**TWC** = Thomas J. Wood Collection, Mons, Belgium

**ICC** = Ian Cross Collection, Briantspuddle, Dorset, United Kingdom

In the text, the following abbreviations are used for morphological terms:

- F1, F2, F3 = flagellomeres 1, 2, 3
- MOD = anterior ocellar diameter
- MS = malar space, the shortest distance between base of mandible and margin of compound eye
- OOL = oculo-ocellar line, the shortest distance between lateral ocellus and compound eye
- P = pedicel
- PD = puncture diameter
- POL = the shortest distance between posterior ocelli
- T1–T3 = metasomal terga 1 to 3
- vs. = versus

Photographs were taken with a Camera Olympus E-M1 Mark II with the Olympus Zuiko 60 mm objective and stacked with the software Helicon Focus (ver. 7.6). Further image processing was completed with Adobe Photoshop CS6.0.

## Data resources

The InBIO Barcoding Initiative Database: contribution to the knowledge on DNA barcodes of "European Chrysididae" dataset can be downloaded from the Public Data Portal of BOLD (http://dx.doi.org/10.5883/DS-IBIHY02) in different formats (data as dwc, xml or tsv and sequences as fasta files). Alternatively, BOLD users can log-in and access the dataset via the Workbench platform of BOLD. All records are also searchable within BOLD, using the search function of the database.

The version of the dataset, at the time of writing the manuscript, is included as Suppl. material 1 in the form of one text file with specimen data information, as Suppl. material 2 in the form of DWC file specimen data and one fasta file containing all sequences as downloaded from BOLD (Suppl. material 3).

## Taxon treatments

### Hedychridium calcarium

Rosa sp. nov.

3F0C3582-68A5-56F4-AB54-560BFAF22973

3F4762B4-7EAB-4B8F-9B63-F18EA15255C4

#### Materials

- **Type status:** Holotype. **Occurrence:** catalogNumber: INV12794; recordNumber: INV12794; recordedBy: Thomas Wood; individualID: INV12794; individualCount: 1; lifeStage: Adult; otherCatalogNumbers: IBIHM1195-22; occurrenceID: 0D71E997-8420-5092-B584-2E6619F2EA61; **Taxon:** scientificName: Hedychridium sp.; phylum: Arthropoda; class: Insecta; order: Hymenoptera; family: Chrysididae; genus: Hedychridium; **Location:** country: Spain; locality: Sierra de Baza, Prados del Rey; decimalLatitude: 37.375; decimalLongitude: -2.854; **Identification:** identifiedBy: Paolo Rosa; **Event:** year: 2021; month: 6; day: 25; **Record Level:** institutionCode: Universite de Mons
- **Type status:** Paratype. **Occurrence:** catalogNumber: INV12795; recordNumber: INV12795; recordedBy: Thomas Wood; individualID: INV12795; individualCount: 1; lifeStage: Adult; otherCatalogNumbers: IBIHM1196-22; occurrenceID: DF256B6E-111E-51B6-8356-0E121E5033F4; **Taxon:** scientificName: Hedychridium sp.; phylum: Arthropoda; class: Insecta; order: Hymenoptera; family: Chrysididae; genus: Hedychridium; **Location:** country: Spain; locality: Noguera de Albarracin, Barranco de la Olmeda; decimalLatitude: 40.462; decimalLongitude: -1.614; **Identification:** identifiedBy: Paolo Rosa; **Event:** year: 2021; month: 6; day: 27; **Record Level:** institutionCode: Universite de Mons
- **Type status:** Paratype. **Occurrence:** catalogNumber: INV12796; recordNumber: INV12796; recordedBy: Thomas Wood; individualID: INV12796; individualCount: 1; lifeStage: Adult; otherCatalogNumbers: IBIHM1197-22; occurrenceID: D746B3CE-8112-585E-B5AA-FBB5866002CB; **Taxon:** scientificName: Hedychridium sp.; phylum: Arthropoda; class: Insecta; order: Hymenoptera; family: Chrysididae; genus: Hedychridium; **Location:** country: Spain; locality: Villar del Cobo, Barranco de los Oncenachos; decimalLatitude: 40.397; decimalLongitude: -1.674; **Identification:** identifiedBy: Paolo Rosa; **Event:** year: 2021; month: 6; day: 19; **Record Level:** institutionCode: Universite de Mons

#### Description

**Figure 3.**
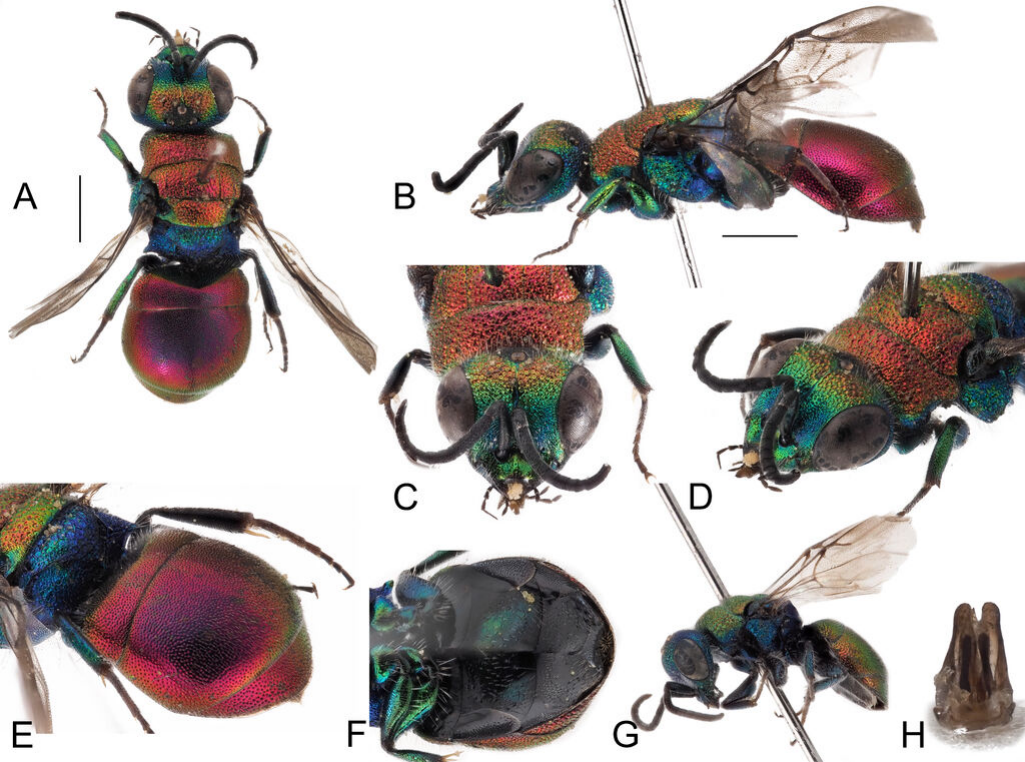
*Hedychridiumcalcarium* sp. n., A-F holotype, female. **A** habitus, dorsal view; **B** habitus, lateral view; **C** head, frontal view; **D** head and mesosoma, fronto-lateral view; **E** metasoma, postero-lateral view; **F** metasoma, ventral view; **G** parataype male, habitus, lateral view; **H** genital capsule. Scale bar: 1 mm.

*Female*. Body length 5.0–5.4 mm (holotype 5.4 mm (Fig. 3)). Forewing length 3.0–3.5 mm.

*Head*. Brow with medium, contiguous punctures (ca. 0.4 × MOD), suddenly decreasing diameter from frontal declivity to malar spaces and clypeus (Fig. 3C); face, in frontal view, micropunctate along inner eye margin; scapal basin with polished intervals; medial line complete from anterior ocellus to clypeus; clypeus finely punctate with wide polished intervals; clypeal apical margin thickened, triangularly-shaped, non-metallic brown; ocellar area with small punctures, without line connecting posterior ocelli; temples regularly rounded, double punctate. OOL = 1.9 × MOD; POL = 1.5 × MOD; MS = 0.6 × MOD; relative length of P:F1:F2:F3 = 1:1.1:0.8:0.8. Malar space as long as antennal thickness.

**Figure 4.**
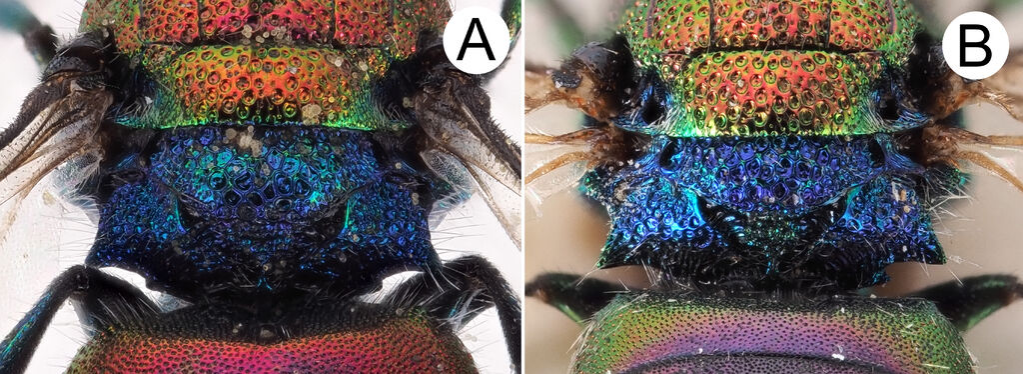
Scutellum, metanotum, metapectal-propodeal complex **A** *Hedychridiumcalcarium* **sp. nov.**, holotype, female; **B** *Hedychridiumjucundum*, female, from Italy (PRC).

*Mesosoma*. Pronotum with punctation irregularly sized, mostly contiguous and large, umbelicate punctures up to 0.6 MOD; intervals between large punctures densely micropunctate. Mesoscutum with similar punctuation, yet punctures relatively smaller and micropunctures sparser compared to intervals on pronotum. Scutellum with polished intervals and sparse micropunctures. Metascutellum with reticulate-foveate punctures (0.8 × MOD). Metapectal-propodeal complex with metapostnotum wider than in other species (Fig. 4A) and posterior propodeal projection [= propodeal teeth] triangular, with thickened, blunt apex, slightly pointing backwards. Forewing medial vein 1.5 times as long as RS stub, medially gently arched; Rs stub as along as pterostigma. Hind leg unmodified, metatibia entirely black, without visible spots or depressions.

*Metasoma*. Punctation on terga minute, even, sparse, regularly spaced, 1–2 PD apart. Third tergum laterally with denser, deeper punctures (Fig. 3E); posterior margin with hyaline rim (2 PD).

*Colouration*. Head blue with two large golden-red spots on brow, between anterior ocellus and eyes; clypeus, malar space and base of mandible greenish; ocelli area blackish. Pronotum and mesonotum red, lateral and posterior margin of scutellum green; rest of mesosoma blue, with green mesopleuron and legs. Metasoma dorsally red, ventrally black with two large, oblique green to blue spots on second sternum (Fig. 3C). Mandible entirely dark brown. Scape black with slight metallic reflection, pedicel and flagellomeres black; tegula black. Wings slightly infuscate.

*Male*: Paratype from Teruel similar to female, with face laterally covered with appressed, silvery setae; antennae elongate, with slender flagellum and cylindrical articles. Paratype from Granada smaller (4.0 mm) with red colouration turned to green (Fig. 3G); genital capsule as in Fig. 3H, with slender cuspis, apically unmodified.

#### Diagnosis

The genus *Hedychridium* Abeille de Perrin, 1878 in Iberia includes 34 species and two subspecies (Rosa and Soon 2012), whereas three previous members were recently moved from the genus *Hedychridium* to the genus *Colopopyga* Semenov-Tian-Shanskji, 1954 (Rosa 2017). Twenty-five of these species are known from Portugal (Rosa et al., in preparation). Mingo (1994) listed only 26 species for Iberia, another nine were overlooked, but has previously been described or cited from Spain by Linsenmaier (Linsenmaier 1959, Linsenmaier 1968, Linsenmaier 1987) and two species, *H.infantum* Linsenmaier, 1997 and *H.balearicum* Strumia, 2013, were described later.

The list of *Hedychridium* species known from Iberia is given below, with species subdivided by species groups following Linsenmaier’s classification (Rosa et al. 2022) and modifications proposed by Pauli et al. (2019), based on multigene molecular analyses.

*anale* group: *Hedychridiumanale* (Dahlbom, 1854); *H.dubium* Mercet, 1904;

*ardens* group: *H.adventicium* Zimmermann, 1961, *H.aereolum* du Buysson, 1891, *H.ardens* (Coquebert, 1801), *H.buyssoni* Abeille de Perrin, 1887, *H.cupritibiale* Linsenmaier, 1987, *H.ibericum* Linsenmaier, 1959, *H.infans* Abeille de Perrin, 1879, *H.infanssantschii* Trautmann, 1927, *H.infantum* Linsenmaier, 1987, *H.jucundum* (Mocsáry, 1889), *H.marteni* Linsenmaier, 1951; *H.reticulatum* Abeille de Perrin, 1878, *H.sevillanum* Linsenmaier, 1968;

*coriaceum* group: *H.coriaceum* (Dahlbom, 1854), *H.krajniki* Balthasar, 1953;

*cupratum* group: *H.cupratum* (Dahlbom, 1854);

*femoratum* group: *H.elegantulum* du Buysson, 1887, *H.femoratum* (Dahlbom, 1854), *H.gratiosum* Abeille de Perrin, 1878;

*heliophilum* group: *H.heliophilum* du Buysson, 1887, *H.vachali* Mercet, 1915;

*incrassatum* group: *H.incrassatum* (Dahlbom, 1854);

*monochroum* group: *H.balearicum* Strumia, 2013, *H.carmelitanum* Mercet, 1915, *H.minutussimum* Mercet, 1915, *H.monochroum* du Buysson, 1888;

*plagiatum* group: *H.andalusicum* Trautmann, 1920, *H.franciscanum* Linsenmaier, 1987;

*roseum* group: *H.chloropygum* du Buysson, 1888, *H.mediocrum* Linsenmaier, 1987, *H.roseum* (Rossi, 1790), *H.scutellare* (Tournier, 1878), *H.subroseum* Linsenmaier, 1959, *H.subroseumprochloropygum* Linsenmaier, 1959.

The following three species were moved in the genus *Colpopyga*, supported by morphological and molecular evidence (Rosa 2017, Pauli et al. 2019): *C.auriventris* (Mercet, 1904), *C.flavipes* (Eversmann, 1858) and *C.temperata* (Linsenmaier, 1959). Three species are also known for the Canary slands and are considered endemic: *Hedychridiumextraneum* Linsenmaier, 1993, *H.tricavatum* Linsenmaier, 1993 and *H.viridicupreum* Linsenmaier, 1993.

Another species, *Hedychridiumsuave* (Tournier, 1878), was described from Spain (Andalucía) and has been considered to be a synonym of *H.roseum* by Linsenmaier (1951), Mingo (1994) and Kimsey and Bohart (1991), who erroneously placed the type locality in Switzerland (Léman area). None of these authors examined the type deposited at the Museum in Geneva. According to the labels pinned with the type specimen, the type locality is Tangier in Morocco and not Andalucía. *Hedychridiumsuave* does not belong to the *roseum* group, but to the *femoratum* group; it is a valid species and, based on its aspect and colouration, this taxon should be a North African species and the Andalusian locality is an error. Tournier is well-known for confusing European and Moroccan localities, as has already happened in other insect families as well as in Chrysididae (see the case of *Chrysissuperba* Tournier, 1879 in Linsenmaier (1968). For the moment, we do not consider *H.suave* to be a member of the Iberian fauna.

*Hedychridiumcalcarium* sp. nov. belongs to the *ardens* species group due to the shape of the second metatarsomere which is longer than the third, the punctate scapal basin, the general habitus and the body colouration (Fig. 3). *Hedychridiumcalcarium* sp. nov. has small to medium dimensions, from 4.0 to 5.4 mm; head blue with two red patches on brow between anterior ocellus and compound eye; black ocellar area; red pronotum and mesonotum, rest of mesosoma blue with greenish reflections; metasoma dorsally red and ventrally black with two large and oblique green-bluish spots on the second sternum. Punctation dense, even and deep on vertex; the largest punctures deep and umbelicate on pronotum, with intervals densely micropunctate; mesoscutum with smaller, shallower and sparser punctures, intervals less densely micropunctate compared to pronotum; metanotum with sparse micropunctures on shining intervals; metapostnotum distinctly enlarged compared to the same morphological part of the closest species, *H.jucundum* (Fig. 4), in which it is triangular. Metasomal sculpture with even, dense and small punctures equally spaced; apical margin of the third tergum with wide hyaline margin (2–3 PD).

Besides different body sculpture and morphological characters, *Hedychridiumcalcarium* sp. nov. can be immediately separated from *H.ardens*, *H.marteni* and *H.ibericum* by its blue metanotum, contrasting with red scutellum (concolourous in the other species); from *H.cupritibiale* by the blue face, contrasting with the red head on vertex (entirely red in *H.sevillanum*); from *H.sevillanum* by the different body colour, which is green to bronze in the latter and by the metanotum bronze to green, slightly contrasting with the rest of the red body colour. For comparison, pictures of *H.ardens* can be found in Paukkunen et al. (2015) and pictures of all the remaining species can be found in the illustrated catalogue of Linsenmaier’s types (Rosa et al. 2022). Finally, *H.infans*, *H.adventicium* and *H.infantum* can be immediately separated by their very small size (2–3 mm) and the different colouration, the first having metallic tegulae (a unique feature), the other two a green line along the posterior margin of the pronotum. The species morphologically and chromatically closer to *H.calcarium* sp. nov. are *H.jucundum*, *H.reticulatum* sensu Linsenmaier (1959) and *H.buyssoni*. However, *H.jucundum* can be differentiated by a dark to black spot on discum of second tergum and by the vertex entirely golden to red; in case of doubt, the triangular shape of the metapostnotum is diagnostic (Fig. 4); *H.reticulatum* by the red mesopleuron and, finally, *H.buyssoni* by the green vertex, the stocky body, the first tergum shorter medially and angled on anterior margins, the metasoma with denser and deeper punctures. The male of *H.calcarium* sp. nov. has the same colouration of the female and can be separated from the similar male of *H.jucundum* by the colour of the head and the shape of the genital capsule with cuspis apically slender, unmodified (vs. apically enlarged and curved in *H.jucundum* (see Rosa 2017)).

Outside the Iberian Peninsula, only *Hedychridiumbytinskii* Linsenmaier, 1959 can be confused with *H.calcarium* sp.nov. *H.bytinskii* was described from Palestine and is known from Greece and Turkey (Linsenmaier 1968, Linsenmaier 1999). Linsenmaier (Linsenmaier 1968, Linsenmaier 1999) listed this species from Morocco, but the Moroccan specimens may actually belong to the western Mediterranean species *H.calcarium*. The latter can be immediately recognised by the dark metasomal sterna, with two small dark green spots on the second sternum, whereas *H.bytinskii* specimens from the east Mediterranean have the first sternum (largely) and second sternum (entirely) bright green (see pictures of the type in Rosa et al. (2022), Fig. 7E). The second sternum of *H.bytinskii* is also characterised by only a few and sparse punctures bearing long setae, whereas *H.calcarium* has a denser punctation (Fig. 1F) with short setae that are approximately one third as long as those of *H.bytinskii*. The colour pattern of the head also differs between the two species, with the entire vertex in *H.bytinskii* coloured flame red, distinctly contrasting the blue head and the green declivity of the frons, with the ocelli area flame red; the red area on the vertex of *H.calcarium* is less strongly contrasting and the ocelli area is black. The scutellum is entirely flame red in *H.bytinskii*, whereas it is metallic green on its posterior margin in *H.calcarium*. Barcoding analyses of the eastern Mediterranean *H.bytinskii* are needed to evaluate the genetic distance between the two species.

##### Genetics

**Figure 5.**
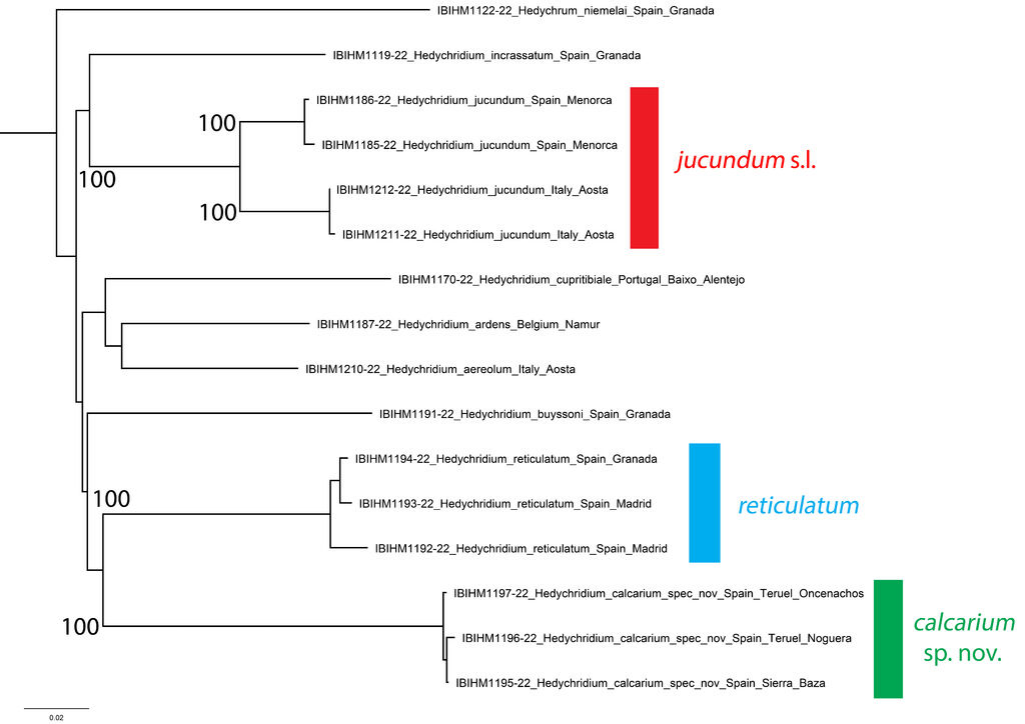
Phylogenetic tree (neighbour-joining) of members of the *Hedychridiumardens* group based on the DNA barcoding mitochondrial COI gene fragment. Numbers adjacent to branches represent bootstrap support (values of < 0.75 are omitted). The scale-bar indicates the % of sequence divergence.

*Hedychridiumcalcarium* sp. nov. is very distinct genetically (Fig. 5), showing a low average intraspecific genetic distance of 0.30%. It is strongly separated from the nearest relative in the phylogenetic tree *H.reticulatum* by an average genetic distance of 16.45% (range 16.11-16.72%). By direct genetic distance, it is closest to *H.jucundum* specimens from Italy, separated by an average of 16.13% (range 15.96-16.41%). As a note, *H.jucundum* specimens from Italy are separated from *H.jucundum* specimens from Menorca by an average of 4.94% (range 4.71-5.17%). This requires further investigation.

#### Etymology

The epithet *calcarium* derives from the Latin adjective *calcarius* related to the limestone habitat of the species.

#### Distribution

**Figure 6.**
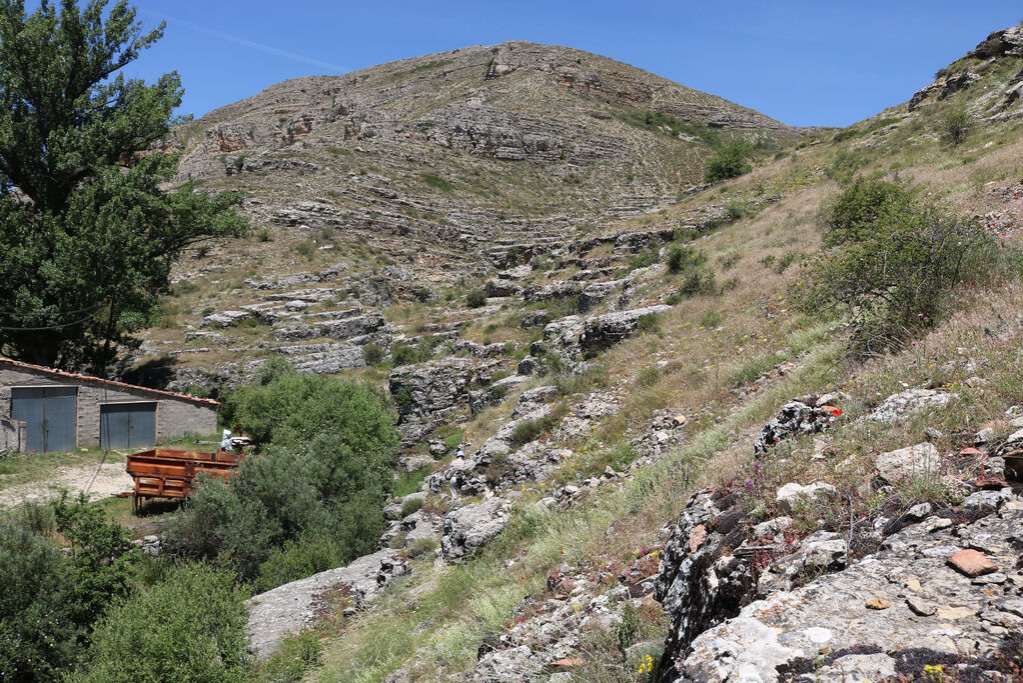
Villar del Cobo, Barranco de los Oncenachos, province of Teruel, Spain. Collecting site for *Hedychridiumcalcarium* Rosa **sp. nov.**

Spain (provinces of Teruel and Granada). At each locality, the species was found in dry grassland on calcareous soil, such as at the Barranco de los Oncenachos (Fig. 6).

#### Ecology

The host is unknown, but is likely to be a small apoid wasp, in line with other members of the *Hedychridiumardens* group.

### Chrysis crossi

Rosa sp. nov.

327DFD24-8176-5011-B6AA-499932314EA2

759C3BE4-F1BE-47B2-9C43-0FFD17612E43

#### Materials

- **Type status:** Holotype. **Occurrence:** catalogNumber: INV12727; recordNumber: INV12727; recordedBy: I. C. Cross; individualID: INV12727; individualCount: 1; sex: F; lifeStage: Adult; otherCatalogNumbers: IBIHM1128-22; occurrenceID: 6CF2BF38-8154-53D2-AB88-640F8CDD5821; **Taxon:** phylum: Arthropoda; class: Insecta; order: Hymenoptera; family: Chrysididae; genus: Chrysis; specificEpithet: crossi; **Location:** country: Portugal; locality: Salema; decimalLatitude: 37.06; decimalLongitude: -8.83; **Identification:** identifiedBy: Paolo Rosa; **Event:** year: 2017; month: 4; day: 16; **Record Level:** institutionCode: Universite de Mons
- **Type status:** Paratype. **Occurrence:** recordedBy: J. Smit (JSC); individualCount: 1; sex: M; lifeStage: Adult; occurrenceID: 9849F20C-7561-5544-B4E2-430469C8A89F; **Taxon:** phylum: Arthropoda; class: Insecta; order: Hymenoptera; family: Chrysididae; genus: Chrysis; specificEpithet: crossi; **Location:** country: Portugal; locality: 3 km N Mexilhoeira Grande, Poio; decimalLatitude: 37.2; decimalLongitude: -8.6; **Identification:** identifiedBy: Paolo Rosa; **Event:** year: 2005; month: 4; day: 30
- **Type status:** Paratype. **Occurrence:** recordedBy: M. & E. Howe (PRC); individualCount: 2; sex: M; lifeStage: Adult; occurrenceID: 01CC81B6-FEC3-5EBD-B89E-B7195661A5AD; **Taxon:** phylum: Arthropoda; class: Insecta; order: Hymenoptera; family: Chrysididae; genus: Chrysis; specificEpithet: crossi; **Location:** country: Portugal; locality: Carrapateira, Praia da Bordeira; decimalLatitude: 37.2; decimalLongitude: -8.9; **Identification:** identifiedBy: Paolo Rosa; **Event:** year: 2006; month: 4; day: 16
- **Type status:** Paratype. **Occurrence:** recordedBy: I.C. Cross (ICC); individualCount: 1; sex: M; lifeStage: Adult; occurrenceID: 92FDD0A6-54DE-5166-B4DF-372F55037E84; **Taxon:** phylum: Arthropoda; class: Insecta; order: Hymenoptera; family: Chrysididae; genus: Chrysis; specificEpithet: crossi; **Location:** country: Portugal; locality: Carrapateira; decimalLatitude: 37.2; decimalLongitude: -8.9; **Identification:** identifiedBy: Paolo Rosa; **Event:** year: 2016; month: 4; day: 26
- **Type status:** Paratype. **Occurrence:** recordedBy: P. Rosa & M. Jacobs; individualCount: 1; sex: M; lifeStage: Adult; occurrenceID: 66AAAE02-1293-52EB-8267-4DBC53119F00; **Taxon:** phylum: Arthropoda; class: Insecta; order: Hymenoptera; family: Chrysididae; genus: Chrysis; specificEpithet: crossi; **Location:** country: Portugal; locality: Estoi; decimalLatitude: 37.1; decimalLongitude: -7.9; **Identification:** identifiedBy: Paolo Rosa; **Event:** year: 2019; month: 5; day: 4
- **Type status:** Paratype. **Occurrence:** recordedBy: P. Rosa & M. Jacobs (MJC); individualCount: 2; sex: M; lifeStage: Adult; occurrenceID: 23B773FC-C44D-5C19-AE88-3CB26CCF3BA4; **Taxon:** phylum: Arthropoda; class: Insecta; order: Hymenoptera; family: Chrysididae; genus: Chrysis; specificEpithet: crossi; **Location:** country: Portugal; municipality: Faro; locality: Montenegro; decimalLatitude: 37.0; decimalLongitude: -7.9; **Identification:** identifiedBy: Paolo Rosa; **Event:** year: 2019; month: 5; day: 9
- **Type status:** Paratype. **Occurrence:** recordedBy: M. Jacobs (MJC); individualCount: 6; sex: 5 M, 1 F; lifeStage: Adult; occurrenceID: 4A481E5E-5CD0-5D06-A882-CA7D5B027CEC; **Taxon:** phylum: Arthropoda; class: Insecta; order: Hymenoptera; family: Chrysididae; genus: Chrysis; specificEpithet: crossi; **Location:** country: Portugal; locality: Rocha; decimalLatitude: 37.1; decimalLongitude: -8.5; **Identification:** identifiedBy: Paolo Rosa; **Event:** year: 2021; month: 5; day: 26

#### Description

*Female*. Body length (holotype) 5.0 mm. Forewing length 3.5 mm.

**Figure 7.**
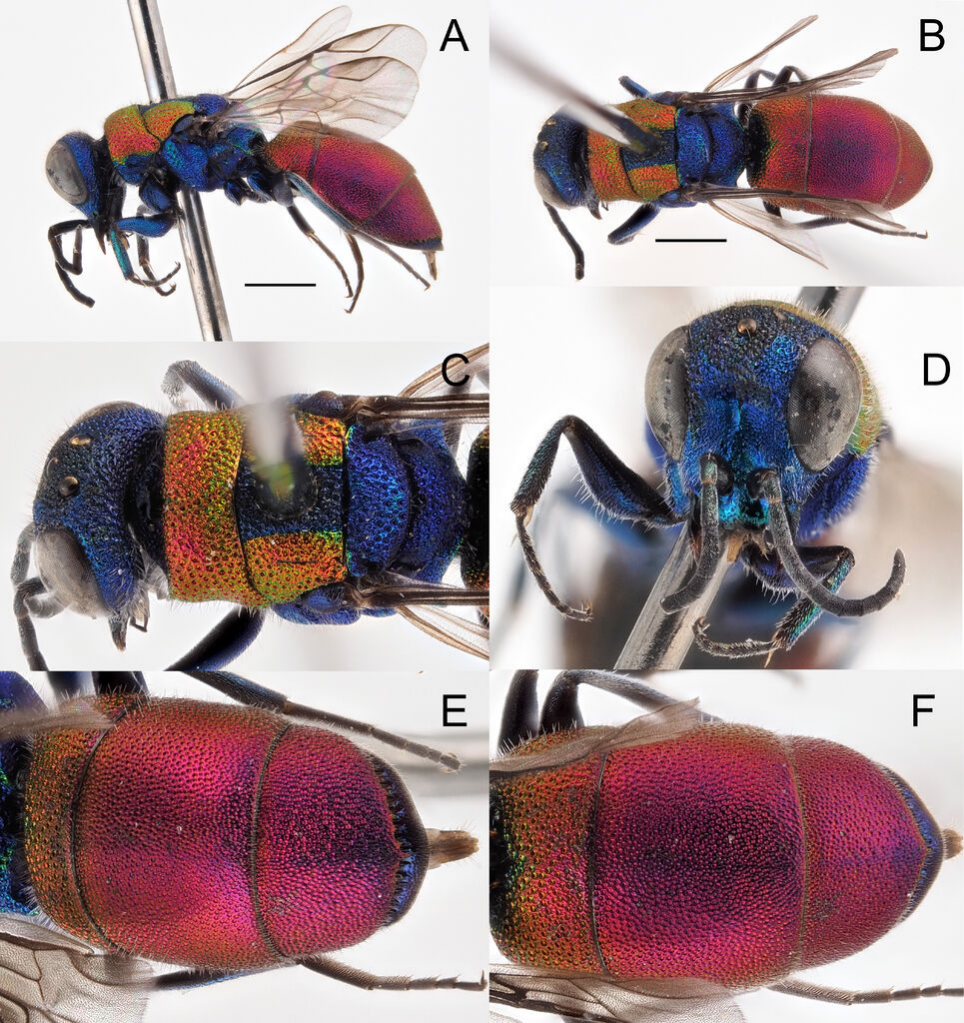
*Chrysiscrossi* Rosa, sp. nov., female, holotype. **A** habitus, lateral view; **B** habitus, dorsal view; **C** head and mesosoma, dorsal view; **D** head, frontal view; **E** metasoma, postero-lateral view; **F** metasoma, dorsal view. Scale bar: 1 mm.

*Head*. Vertex and frons with small, contiguous punctures (from 0.2× to 0.3× MOD) and polished interspaces below brow; transverse frontal carina faint; scapal basin medially transversally microridged, laterally with small punctures increasing diameter towards eye (Fig. 7D); malar spaces densely punctate, elongate (1.7× MOD), shorter than first flagellomere (2.0× MOD) and with short dense, silver setae; genal carina fully developed to mandibular insertion; clypeus mostly polished, sparsely punctate along anterior margin; clypeus elongate, subantennal area 1.7 MOD; medially notched and apically thickened. First flagellomere elongate, l/w = 4 (width taken at base of flagellomere). OOL 1.4× MOD; POL 2.0× MOD; MS 1.7× MOD; relative length of P:F1:F2:F3 = 1.0:1.6:0.9:0.7.

*Mesosoma*. Medial pronotal line narrow and short, reaching half pronotal length; pronotum antero-laterally slightly bulging (Fig. 7); pronotal punctation double and interspaces polished with sparse minute dots; notaulus basally formed by small subrectangular foveae becoming smaller and rounded at apex; parapsidal signum as a linear depression; mesoscutellum dense puncture and irregular interspaces, antero-medially corrugated and becoming polished towards base; scrobal sulcus of mesopleuron formed by large foveae aligned, limited to upper half; episternal sulcus formed by large and irregular, subsquare foveae; punctation with dots on interspaces and larger punctures on mesepisterum; scutellar-metanotal suture deep and wide; metanotum with contiguous punctures, larger than other punctures on mesosoma; posterior propodeal projections slightly divergent; wing venation unmodified.

*Metasoma*. First tergum double punctate, with large punctures separated by small punctures on interspaces; second and third tergum double punctate, larger punctures smaller than those on first tergum; punctures on metasomal separated by polished interspaces (Fig. 7B, Fig. 7F); pit row composed by small, deep pits, apical margin of third tergum continuous, dark blue, medially arcuate; black spots of the second sternum large, covering almost all segment length, reaching median line.

*Colouration*. Head and mesosoma dark blue, pronotum and lateral areas of mesoscutum flame red, scutellum with light blue highlights; metasoma red to purplish, apical margin of third tergum blue. Scape, pedicel and first tergum black with weak greenish-metallic lustre, rest of flagellum black; tegula blue; metasomal venter black, with only a narrow blue thin line between the black spots and the apical margin of the second sternum. Legs blue, tarsi dark brown.

**Figure 8.**
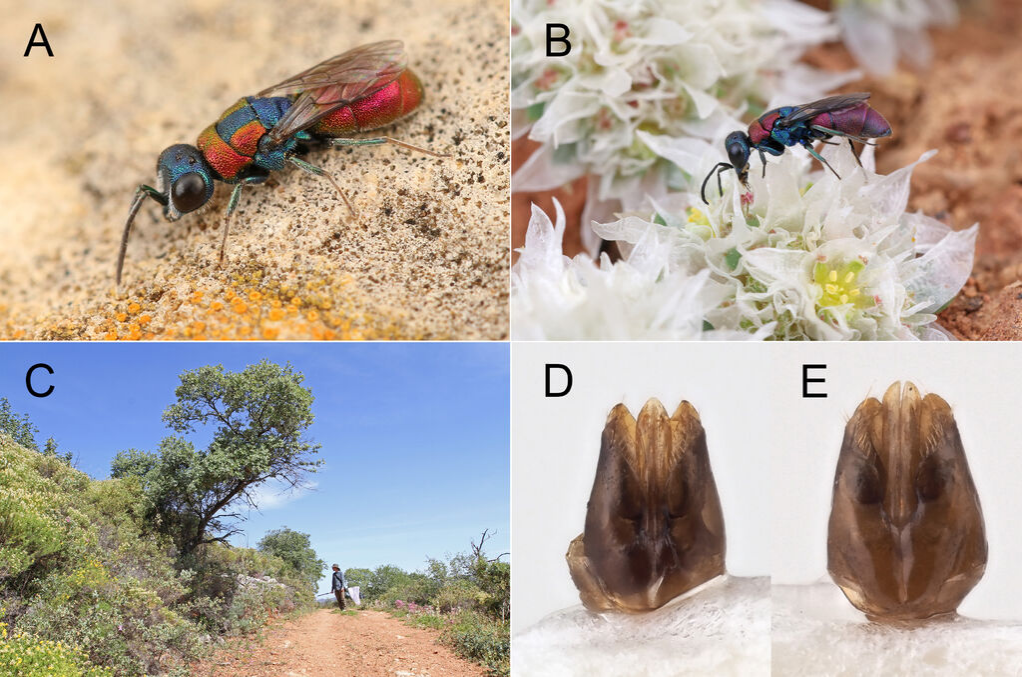
*Chrysiscrossi* Rosa, **sp. nov. A** male (Ph.: © M. Jacobs); **B** female (Ph.: © M. Jacobs); **C** collecting site of *Chrysiscrossi* with Maarten Jacobs; **D** Male genital capsule; **E** Male genital capsule of *Chrysisphryne* from Italy (Emilia-Romagna, Oriano).

*Male*. Body length 5.0–6.0 mm. Similar to female in shape, sculpture and colouration. Malar space slightly shorter, scapal basin laterally covered by short, dense, appressed and silvery pubescence; blue segments of mesososoma with greenish reflection, propodeum and propodeal angles dorsally green to golden green; brown. Male genital capsule (Fig. 8A) with inner margin of the gonocoxa straight.

#### Diagnosis

**Figure 9.**
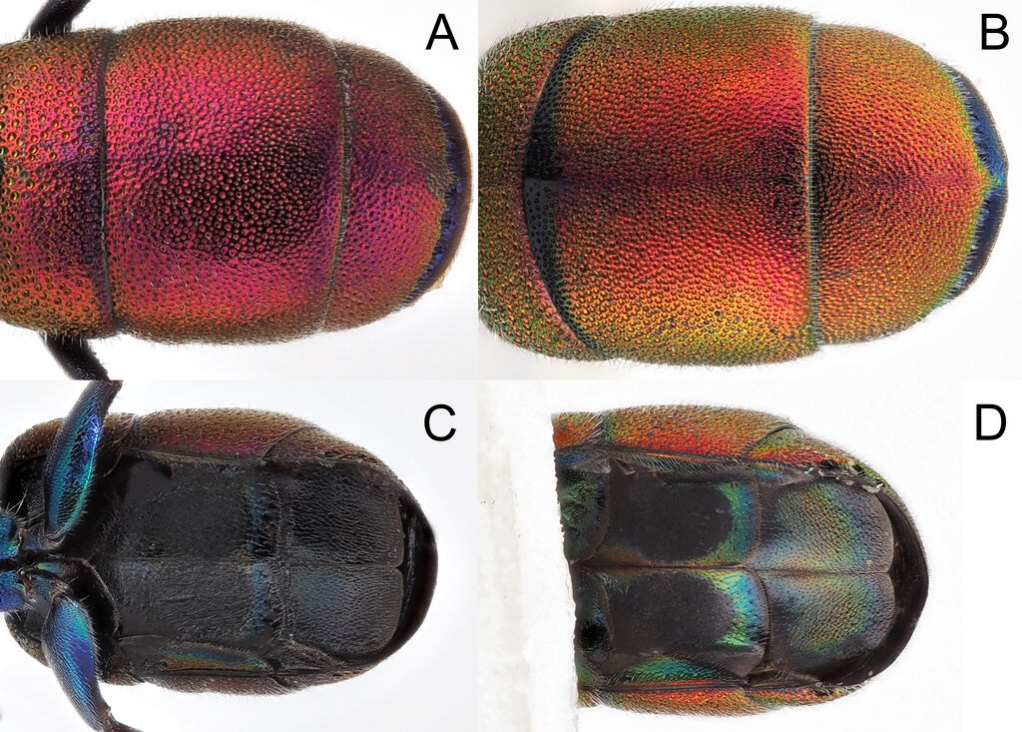
**A-B** metasoma, dorsal view: **A** *Chrysiscrossi* Rosa, sp. nov., male, paratype; **B** *Chrysisphryne* male, from Italy (Emilia-Romagna, Oriano); **C-D** metasoma, ventral view; **C** *Chrysiscrossi* Rosa, sp., nov., male, paratype; **D** *Chrysisphryne* male, from Italy (Emilia-Romagna, Oriano).

Medium-sized, slender species (5–6 mm); head and mesosoma blue, pronotum and lateral areas of mesoscutum red; mesosoma dorsally red to purple, apical margin of third tergum blue; metasoma ventrally black, black spots on second sternum large, covering almost all surface and touching mid-line, without being clearly fused with each other; narrow stripe on apical margin of second tergum blue. Metasoma punctation double, dense, with polished interspaces between the large and small punctures. *Chrysiscrossi* sp. nov. is chromatically and morphologically similar to *C.phryne* Abeille de Perrin, 1878, but it is clearly separated genetically (see below). The main diagnostic characters to separate both sexes from *C.phryne* is the punctation, which consists of distinct double punctures on the metasomal scutum, these being separated by polished interspaces (Fig. 7E, Fig. 9A), whereas in *C.phryne*, the punctation is even and dense, without polished spaces. The metasomal venter is black in both sexes; black spots on second sternum large, covering almost all surface and touching mid-line with a narrow blue line between the black spots and the apical margin of the segment; in *C.phryne*, the sternum is clearly metallic green to golden green, with black spots distinctly separate from mid-line. The male genital capsule of the two species is different (Fig. 8) being narrower and more slender in *C.crossi* sp. nov., with the inner margin of the gonocoxa straight. Male can be also recognised by their brown tarsi, which are pale to yellowish in *C.phryne*.

##### Genetics

*Chrysiscrossi* is very distinct genetically, being separated from an Italian specimen of *C.phryne* by 8.51%. There are no other specimens separated by less than a genetic distance of 10.0%.

#### Etymology

The specific epithet *crossi* (masculine) is dedicated to Ian Cross (Dorchester, Dorset, UK) for his active research on Portuguese Hymenoptera, including cuckoo wasps, many specimens of which were used for the current InBIO Barcoding Initiative work.

#### Distribution

Portugal (Algarve).

#### Ecology

Label information from Ian Cross reports that a male specimen was collected at an aggregation of *Melitturgacaudata* Pérez, 1879 (Andrenidae), on the sand near empty snail shells. *Chrysisphryne* has been reported to attack Osmia (Allosmia) melanura Morawitz, 1871 (see Pauli et al. (2019)), but this cannot be the typical host across much of its range as, in Europe, *O.melanura* is restricted to southern Italy, North Macedonia and southern Ukraine (Müller 2022). It is likely that a different snail shell-nesting O. (Allosmia) is used instead, all known species of O. (Allosmia) using this nesting substrate. The likely host is O. (Allosmia) rufohirta Latreille, 1811 which is widely distributed across Europe and is the only O. (Allosmia) known from Portugal (Müller 2022), being common in the Algarve (Baldock et al. 2018). We suggest that *O.rufohirta* is the likely host of *C.crossi*, though this must be confirmed through direct observations.

#### Notes

According to Linsenmaier (1959), the *phryne* group includes only two species: *Chrysiscirce* Mocsáry, 1889 and *C.phryne* Abeille de Perrin, 1878, with three subspecies *C.phryne* s.str., *C.phrynehebraeica* Linsenmaier, 1959 and *C.phryneburgenlandia* Linsenmaier, 1968. The types of these subspecies have been examined and *C.hebraeica* **stat. nov.** has to be considered to be a distinct species, based on morphological analyses, as it displays greater morphological differences from *C.phryne* s.str. than *C.crossi*. Comments on the specific status of *C.phryneburgenlandia* (known from Austria to Greece) should be postponed until genetic sequences are available, because the main diagnostic characters are based on body colouration only.

Finally, Mocsáry (1889) described *Chrysisdestefanii*, based on the description of a specimen collected in Sicily by De Stefani-Perez and identified as *C.candens* by du Buysson (1888). The type of *Chrysisdestefanii* is currently considered to be lost, as is large part of De Stefani’s collection (Romano 2006). *Chrysisdestefanii* was considered to be a synonym of *C.phryne* by Linsenmaier (1959) and Kimsey and Bohart (1991). Strumia and Yildirim (2009) identified a specimen from Turkey as *Chrysisdestefanii*, yet this record may be related to *Chrysishebraeica* or to an undescribed species of the similar *rubricata* group that has already been observed in the Middle East (PR, unpublished data). Based on the descriptions by du Buysson (1888) and Mocsáry (1889) and, in particular, on the detail on the punctation of the second tergum “*régulière formée de points égaux, assez serrés*”, the synonymy between *Chrysisdestefanii* and *C.crossi* is excluded and De Stefani’s specimen would appear to be conspecific with *C.phryne*. Since the type of *Chrysisdestefanii* is lost, we treat *C.destefani* as **nomen dubium**, until such a point that molecular data are available for Sicilian specimens.

## Analysis

### Hedychridiumcaputaureum Trautmann & Trautmann, 1919 and Hedychridiumchloropygum du Buysson, 1888

Arens (2010) considered *Hedychridiumcaputaureum* Trautmann & Trautmann, 1919 to be a subspecies of *H.chloropygum* du Buysson, 1888, based on morphological affinities and noticeable variation in surface sculpture. According to Arens (2010), *H.chloropygum* s.str. is limited to south-western Europe, whereas *H.chloropygumcaputaureum* is distributed in northern, central and south-eastern Europe. The main difference between these two taxa is based on the colouration. In *H.chloropygum* s.str., the anterior part of the body is richly adorned with copper or gold and the metasoma is metallic blue to violet on the third tergum, to a varying extent and intensity. In *H.chloropygumcaputaureum*, the anterior part of the body may have a similar colouration, although Nordic and central European specimens may be darker, with faint coppery or golden reflections (described as *H.chloropygumdensum* Linsenmaier, 1959 and synonymised by Arens (2010) with *H.chloropygumcaputaureum*); however, the metasoma is always without metallic reflections. The two taxa can additionally be separated by the denser and coarser punctation of the metasoma in *H.caputaureum* which is apparently locally variable (Arens 2010).

**Figure 10.**
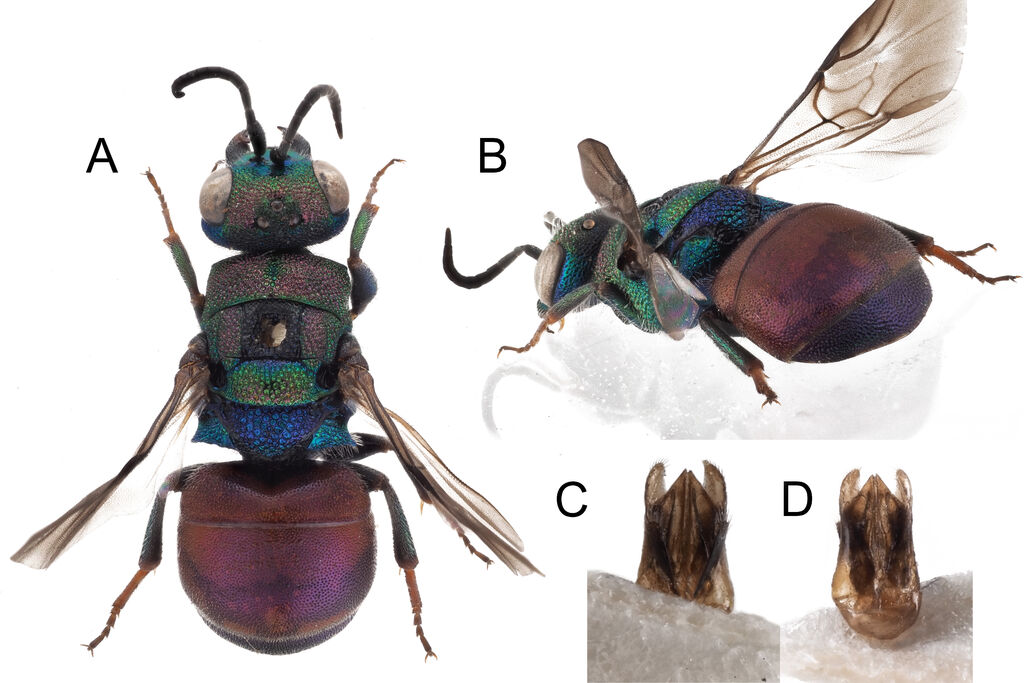
*Hedychridiumcaputaureum* Trautmann & Trautmann, 1919, from Algarve **A** habitus, dorsal view; **B** habitus, postero-lateral view; **C** genital capsule, ventral view; **D** genital capsule, dorsal view.

**Figure 11.**
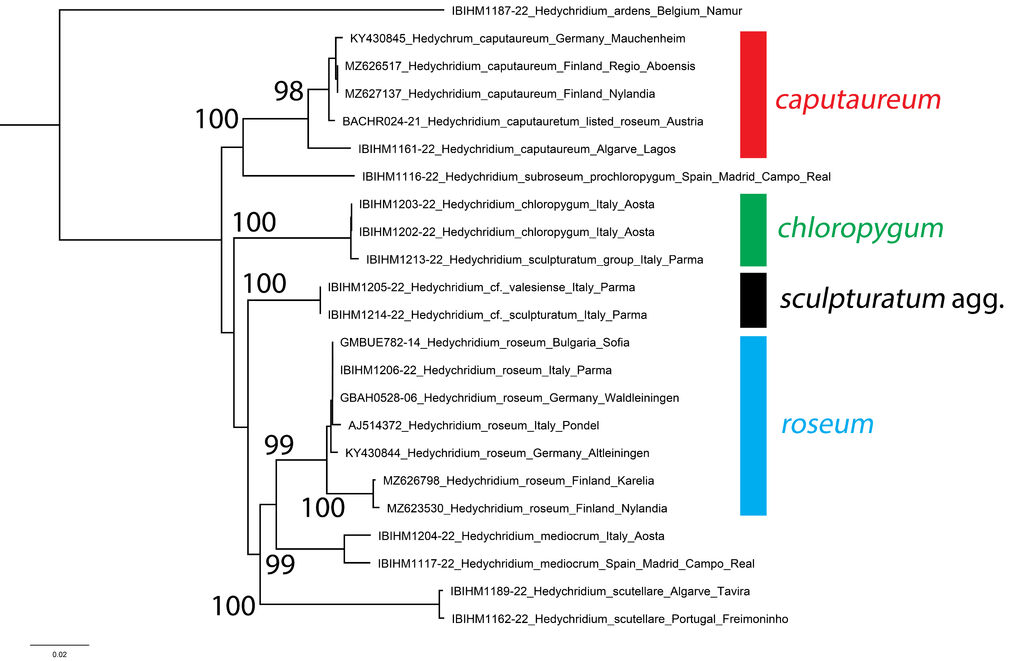
Phylogenetic tree (neighbour-joining) of members of the *Hedychridiumroseum* group, based on the DNA barcoding mitochondrial COI gene fragment. Numbers adjacent to branches represent bootstrap support (values of < 0.75 are omitted). The scale-bar indicates the % of sequence divergence.

The specimen IBIHM1161-22 collected in the Algarve (Lagos, Fig. 10) shows a rich golden colouration of the anterior part of the body and third tergum is entirely violet, with the metasoma densely punctate. DNA barcodes demonstrate there is moderate genetic differentiation between the Portuguese specimen and *H.caputaureum* from northern and central Europe (Fig. 11), being separated by an average of 2.50% (range 2.43-2.59%). The clade of *H.caputaureum* from Austria, Finland and Germany shows low average intraspecific distance of 0.19% (range 0.00-0.34%) and has bootstrap support of 98%.

However, the Portuguese specimen is much more strongly separated from two sequences of *H.chloropygum* from Italy, showing average genetic differentiation of 8.43% (range 8.36-8.51%). When including the Portuguese specimen within *H.caputaureum*, the two clades are separated by an average interspecific genetic distance of 8.56% (range 8.36-9.00%). We, therefore, consider *H.caputaureum* and *H.chloropygum* to be two different species and include Portuguese material within *H.caputaureum*, with the observed genetic distance considered to be variation, given the geographic distance between southern Portugal and Germany. The overall distribution of *H.caputaureum* must be revised, as this Portuguese specimen is the first reported record of this taxon in south-western Europe. Additional genetic samples from Spain and France are likely to fall between the Portuguese and central/northern European sequences.

The subspecies *H.chloropygumberberiacum* Linsenmaier, 1959 from Algeria and Morocco shows a similar colouration to the Algarve specimen, though it has more extensively metallic violet colouration laterally on the metasoma, but also sparser punctation (Rosa et al. 2022). Genetic analyses are needed to clarify the placement of this taxon, but for the moment, we consider it to be the northern African subspecies of *H.chloropygum*, based on its shallow and sparse punctation.

### Hedychrumrutilans Dahlbom, 1854 and Hedychrumviridiaureum Tournier, 1877 sp. resurr.

*Hedychrumrutilans* Dahlbom, 1854 is one of the most common European cuckoo wasp species, known to be a cleptoparasite of *Philanthus* species (Linsenmaier 1997a). In addition to typical cleptoparasitic behaviour, the female does not have to enter the host nest for ovipositing, but can oviposit directly on the prey (*Apismellifera* Linnaeus) while it is being transported to the nest by the host (Veenendaal 1987, Baumgarten 1995). This species is also known in literature as *H.intermedium* sensu auctorum for an incorrect interpretation of the type materials (Rosa and Xu 2015). Linsenmaier (Linsenmaier 1959, Linsenmaier 1997a, Linsenmaier 1997b) considered three European subspecies, namely: *rutilans* s.str., ssp. viridiaureum Tournier, 1877 and ssp. viridiauratum Mocsáry, 1889.

**Figure 12.**
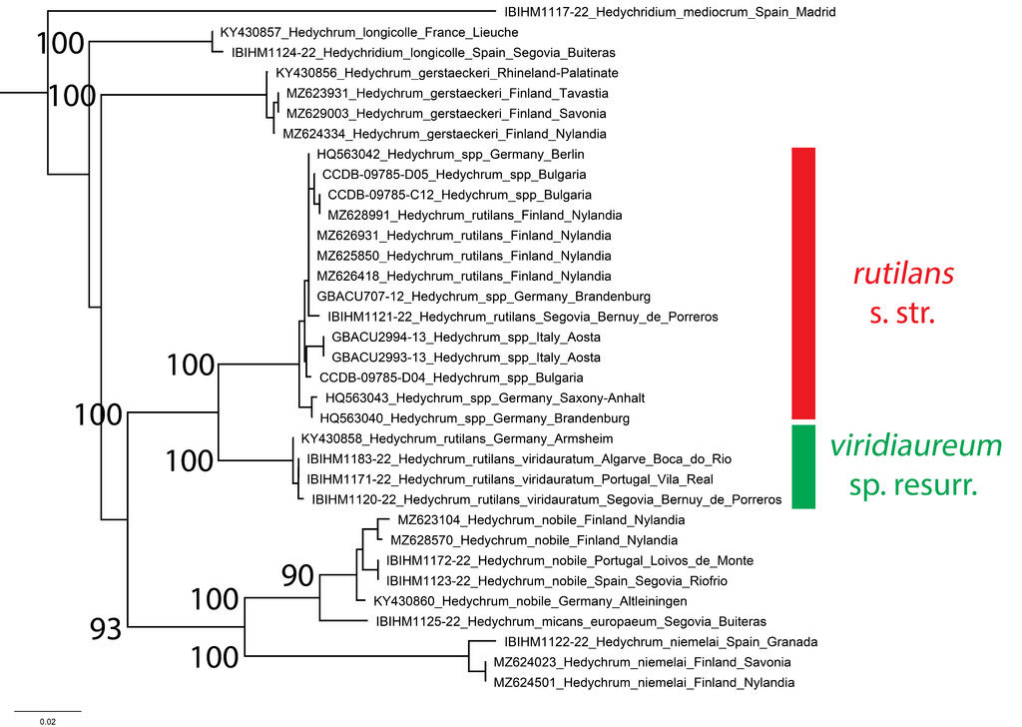
Phylogenetic tree (neighbour-joining) of *Hedychrum* species, based on the DNA barcoding mitochondrial COI gene fragment. Numbers adjacent to branches represent bootstrap support (values of < 0.75 are omitted). The scale-bar indicates the % of sequence divergence.

Two specimens from the same sampling locality in central Spain (Segovia, Bernuy de Porreros, IBIHM1120-22 and IBIHM1121-22) were both identified as *H.rutilans*, but are separated by a genetic distance of 5.27%. Integrating all newly-acquired sequences and sequences from BOLD and GenBank (some without identifications beyond *Hedychrum* sp.) shows that *H.rutilans* s.l. comprises two taxa (Fig. 12). Sequences from Spain, Italy, Germany, Finland and Bulgaria belong to *H.rutilans* s. str. (Fig. 12). They show low average intraspecific distance of 0.55% (range 0.00-1.48) and form a clade with bootstrap support of 100. Sequences from Portugal, Spain and western Germany (Rhineland-Palatinate) also show a low average intraspecific distance of 0.16% (range 0.00-0.33) and have bootstrap support of 100%. The two clades are separated by an average genetic distance of 5.16% (range 4.78-5.77%).

**Figure 13.**
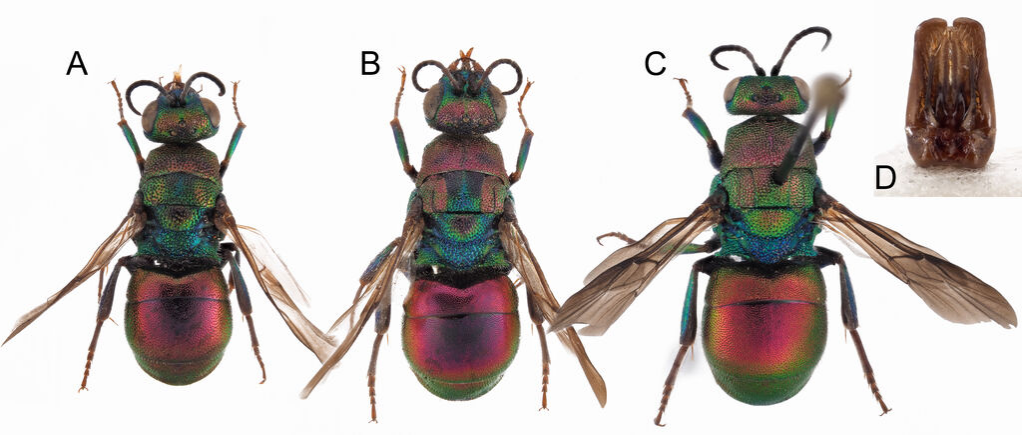
*Hedychrumrutilans* Dahlbom, 1854, habitus, dorsal view **A** male from Austria, Hainburg (NMLU); **B** female from Austria, Hainburg (NMLU); **C** female from Spain, Segovia; **D** male genital capsule, Austria (NMLU).

**Figure 14.**
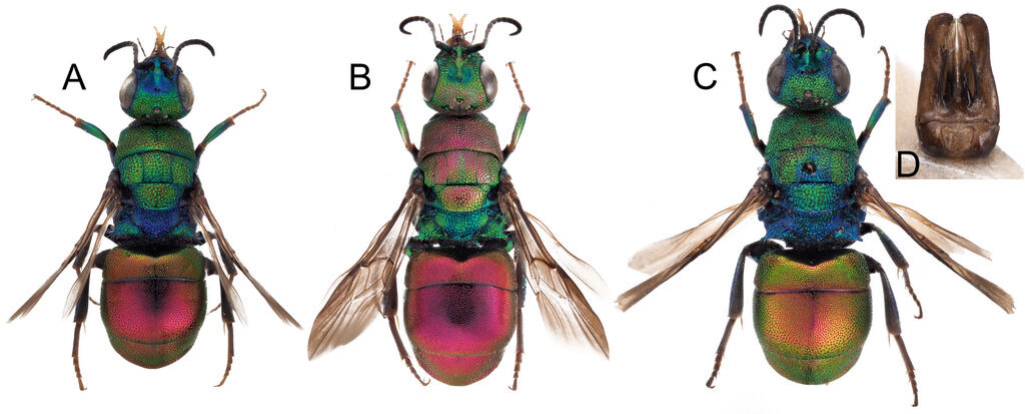
*Hedychrumviridiaureum* Tournier, 1877, habitus, dorsal view **A** male from Switzerland, Wallis (NMLU); **B** female from Spain, Soria (NMLU); **C** male from Spain, Segovia; **D** male genital capsule, Spain, Segovia.

Following Linsenmaier (1997a), the second clade is called *Hedychrumviridiaureum* Tournier, 1877 sp. resurr. Linsenmaier (Linsenmaier 1959, Linsenmaier 1997a) noted differences between these two taxa and employed a subspecific framework. *Hedychrumrutilans* s. str. (Fig. 13) is usually larger and Linsenmaier noted a host association with *Philanthuscoronatus* (Thunberg, 1784), whereas *H.viridiaureum* (Fig. 14) is usually smaller and is associated with the respectively smaller host *Philanthustriangulum* (Fabricius, 1775). Based on the DNA barcodes presented here, combined with the distributional framework of Linsenmaier, in Europe, *H.rutilans* s. str. appears to be more widely distributed, from central Iberia across the continent, whereas *H.viridiaureum* appears to be restricted to Iberia and western Europe, to Switzerland, western Germany and north to Belgium and the Netherlands. Detailed revision is necessary to clarify these range limits and all host associations as, in some regions where *H.rutilans* s.str. occurs, only *P.triangulum* is present. Our observations would support the host associations noted by Linsenmaier, with additional points. Very small individuals of *H.viridiaureum* can be found in Iberia (such as IBIHM-1183-22) where they are associated with *Philanthuspulchellus* Spinola, 1842 that is smaller than *P.triangulum*. Equally, in central Spain, the large-bodied *Philanthusdufourii* Lucas, 1849 is much more frequently encountered than *P.coronatus* and, hence, this is likely the principal host of *H.rutilans* s. str. in this region. The correct placement of *H.rutilansviridiauratum* Mocsáry, 1889, described from Algeria (types examined) and cited by Linsenmaier (Linsenmaier 1959, Linsenmaier 1997a) from the Iberian Peninsula is unclear and must be evaluated by means of molecular analyses. It could be a synonym of *H.viridiaureum* Tournier or a separate northern African species.

### Philoctetespunctulatus (Dahlbom, 1845) and Philoctetesparvulus (Dahlbom, 1845)

**Figure 15.**
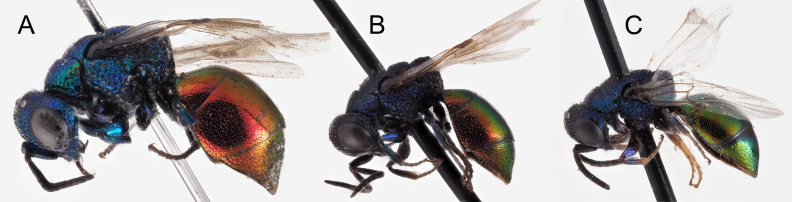
*Philoctetespunctulatus* (Dahlbom, 1854), habitus, lateral view **A** female from Spain, Segovia; **B** female from Portugal, Tavira; **C** male from Portugal, Tavira.

**Figure 16.**
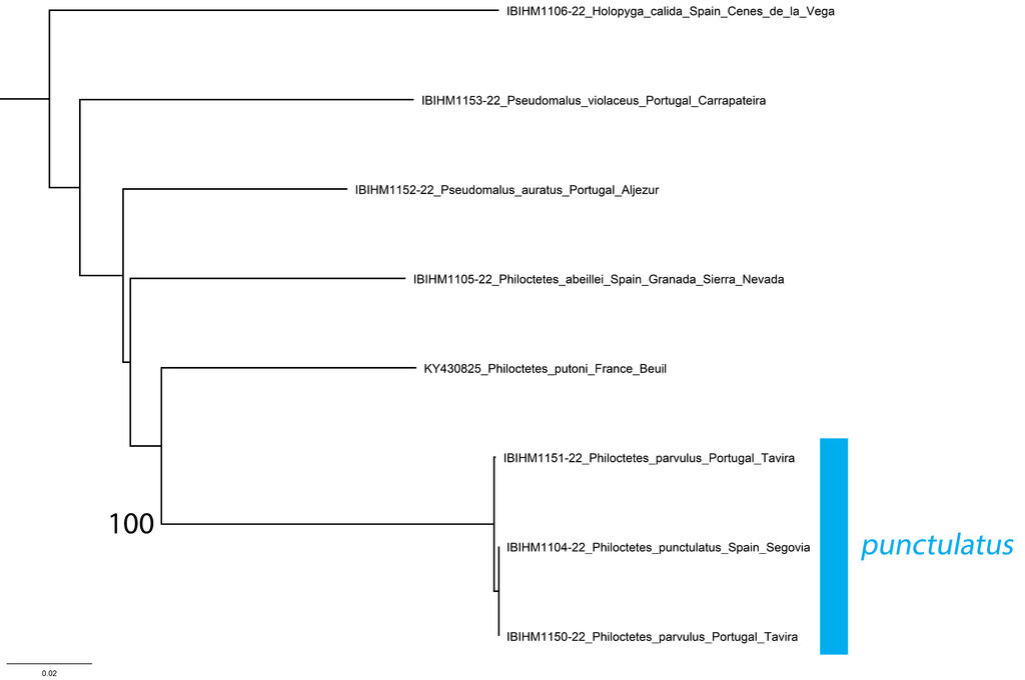
Phylogenetic tree (neighbour-joining) of Elampini species with a focus on *Philoctetes*, based on the DNA barcoding mitochondrial COI gene fragment. Numbers adjacent to branches represent bootstrap support (values of < 0.75 are omitted). The scale-bar indicates the % of sequence divergence.

*Philoctetesparvulus* (Dahlbom, 1845) was considered to be a valid species by Rosa and Soon (2012) following type examination. Based on the consistently smaller size, dark colouration and different punctation of both sexes in comparison with *P.punctulatus* (Fig. 15), specimens of *P.parvulus* were considered to be distinct. However, DNA barcodes (Fig. 16) demonstrate that the small individuals displaying the typical morphology of *P.parvulus* (INV12750, INV12749) show almost no genetic differentiation from *P.punctulatus* (0.00% and 0.02%). The differences in morphology are, therefore, not considered to be species-specific and are probably caused or exaggerated by the smaller body size. *Philoctetesparvulus* is, therefore, considered to be a synonym of *P.punctulatus*.

### Chrysislusitanica Bischoff, 1910

**Figure 17.**
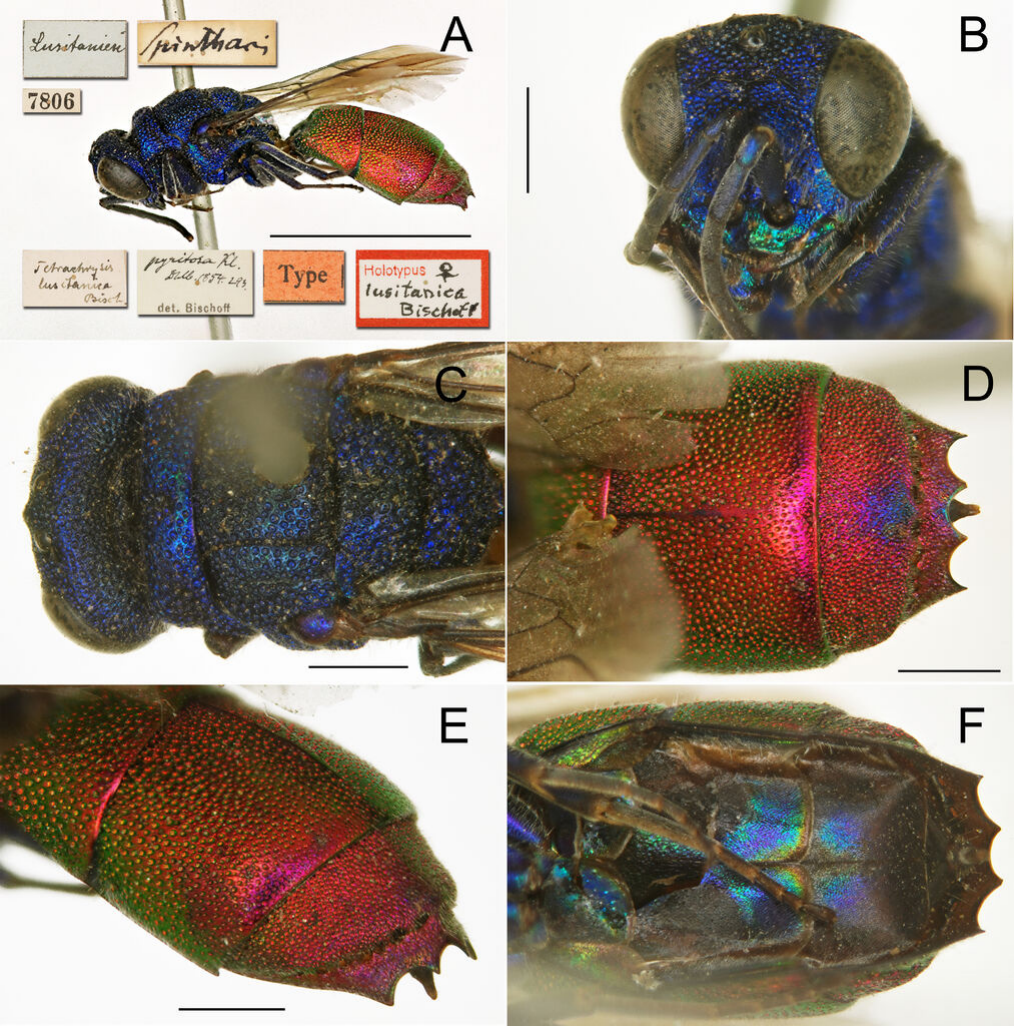
*Chrysislusitanica* Bischoff, 1910, female, holotype (MfN) **A** habitus, lateral view; **B** head, frontal view; **C** head and mesosoma, dorsal view; **D** metasoma, dorsal view; **E** metasoma, postero-lateral view; **F** metasoma, ventral view.

The identity of *Chrysislusitanica* Bischoff, 1910 (Fig. 17) has to date remained unclear. Kimsey and Bohart (1991) synonymised *Chrysissculpturata* Mocsáry, 1912 with *C.lusitanica*, but the examination of both types revealed the occurrence of two distinct species (Rosa et al. 2017, PR, unpublished data). Soon et al. (2014) DNA barcoded and revalidated *C.sculpturata* in the clade of *C.ignita* (Linnaeus, 1758), whereas the placement of *C.lusitanica* remained unknown.

The DNA barcode of a recently-collected specimen places *C.lusitanica* in the clade of *C.brevitarsis* Thomson, 1870, a well-studied group after the molecular works of Soon et al. (2014) and Orlovskyté et al. (2016). In the framework of these previous projects, we can conclude that *C.lusitanica* is a member of the *brevitarsis* clade and that it is also present in Sardinia (first record for Italy, previously published as *C.pseudobrevitarsis* Linsenmaier, 1951 by Soon et al. (2014)). *Chrysislusitanica* can be easily separated from the other species of this clade by the small, even and dense punctures on the second tergum, these punctures being smaller or similar to those on the first tergum and the mesosoma densely punctate, with uniform dark blue colouration.

**Figure 18.**
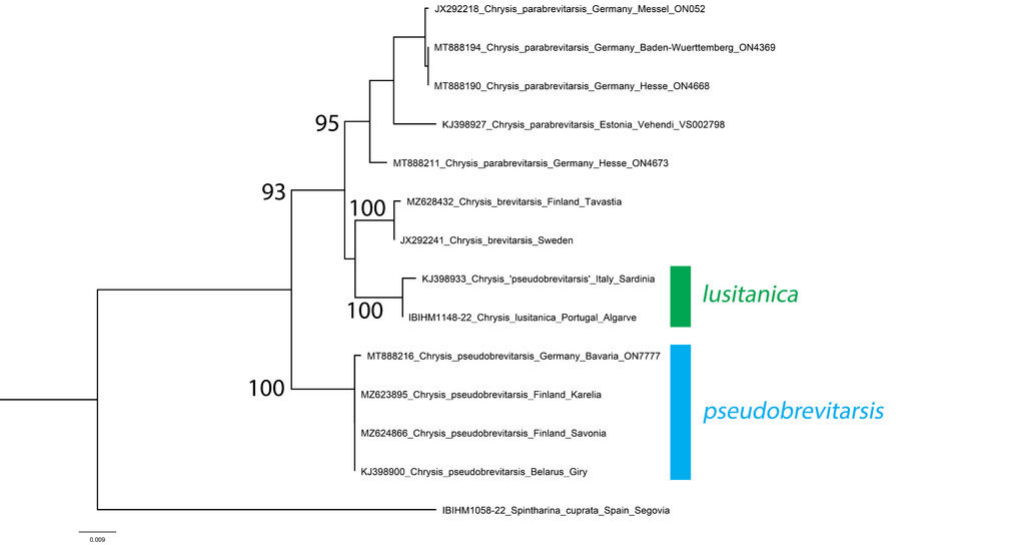
Phylogenetic tree (neighbour-joining) of members of the *Chrysisignita* group with a focus on the species around *Chrysisbrevitarsis* based on the DNA barcoding mitochondrial COI gene fragment. Numbers adjacent to branches represent bootstrap support (values of 0.75 are omitted). The scale-bar indicates the % of sequence divergence.

*Chrysislusitanica* (including the Sardinian specimen that differs from the Portuguese specimen by 0.46%) is strongly separated from *C.pseudobrevitarsis* by an average interspecific distance of 4.07% (range 3.80-4.41%, Fig. 18). It is less strongly separated from *C.brevitarsis* by an average interspecific distance of 2.28% (range 2.05-2.43%), but because intraspecific variation is low (0.46% and 0.16%, respectively), both the *C.lusitanica* and *C.brevitarsis* clades have bootstrap support of 100%. Genetic differentiation within the *brevitarsis* clade is generally low, with *C.brevitarsis* separated from *C.parabrevitarsis* by an average interspecific distance of 3.06% (range 2.46-3.50%). In this context, *C.lusitanica* is considered to be a consistently differentiated species.

### Chrysisscutellarismarteni Linsenmaier, 1951

Linsenmaier (1951) described a Spanish subspecies of *Chrysisscutellaris* Fabricius, 1794, based on its larger size (9.0-10.5 mm) and stocky aspect, with the apical margin of the third tergum indistinctly undulate. This form can be collected in sympatry with the nominotypical species. The two specimens DNA barcoded here conform morphologically to *C.scutellarismarteni* and show a genetic distance of 6.26% and 6.42% from a sample of *C.scutellaris* from Eisenberg (Rhineland-Palatinate) in Germany (KY430717). The subspecies may, therefore, be distinct, but additional samples are required, particularly of typical *C.scutellaris* from Iberia.

### Chrysissplendidula group

The *splendidula* group currently includes twelve Palearctic species, yet the real number of the species in this group is unclear and requires detailed revision. Several morphospecies are found in the Mediterranean region, in particular those related to the subgroup of *C.rutilans* Olivier, 1790 and identified with the name *Chrysisinsperata* Chevrier, 1870, including small and slender species. Other morphospecies closely related to *C.rutilans* are awaiting description (PR, unpublished data). The identity of specimen IBIHM1090-22 from the Sierra Nevada, tentatively identified as *C.rutilans*, is unclear, but it may represent another undescribed taxon within this group as it is clearly separated from *C.rutilans* sequences from Finland.

**Figure 19.**
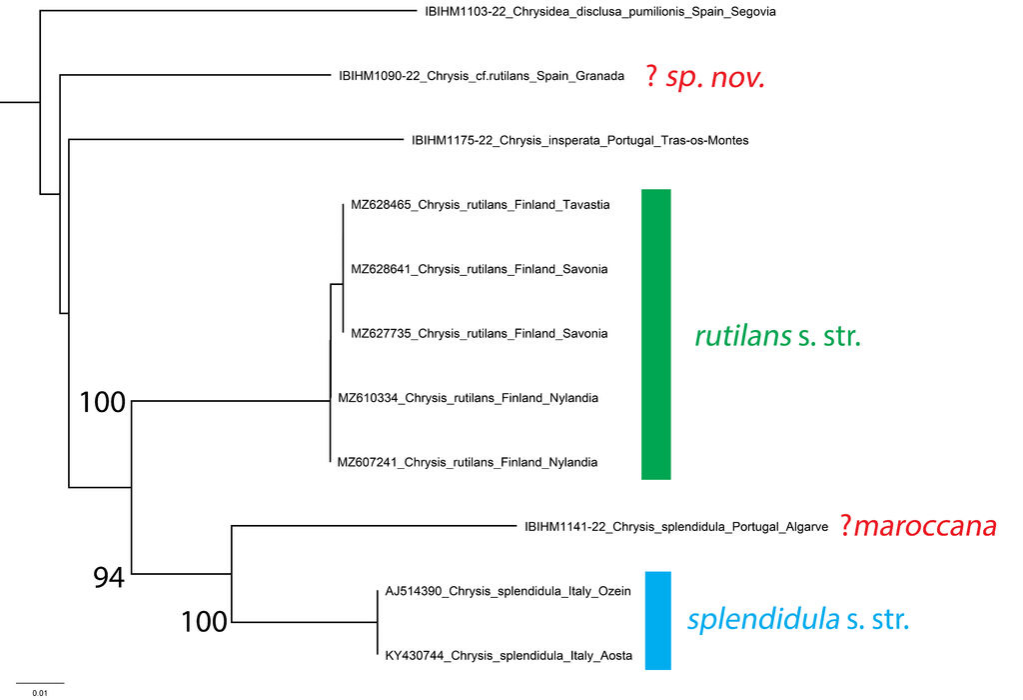
Phylogenetic tree (neighbour-joining) of the *Chrysissplendidula* group, based on the DNA barcoding mitochondrial COI gene fragment. Numbers adjacent to branches represent bootstrap support (values of < 0.75 are omitted). The scale-bar indicates the % of sequence divergence.

Within the *splendidula* group, we identify *Chrysismaroccana* Mocsáry, 1883 as a species closely related to *C.splendidula* Rossi, 1790, which lacks the raised apical margin at the apex of the second tergum. This species was previously reported from Morocco, Sardinia and Corsica (Linsenmaier 1987). Mingo (1994) recorded *C.maroccana* from Portugal and Spain, but her identifications clearly refer to another species already known from Iberia which was not mentioned in the monograph, namely *C.continentalis* Linsenmaier, 1959. Specimen IBIHM1141-22 from southern Portugal (Algarve, Praia do Barril) is strongly separated from two sequences of *C.splendidula* from Italy by an average of 7.67% (range 7.49-7.84%, Fig. 19). We suspect that the Portuguese specimen represents *C.maroccana*, but additional sampling and genetic sequences from Morocco are required for confident determination, given the complexity within this species group

### Stilbumwestermanni Dahlbom, 1845 sp. resurr.

Linsenmaier (1959) listed six subspecies of *Stilbumcalens* (Fabricius, 1781) distributed from Europe to China and he separated the two subspecies *S.calenszimmermanni* Linsenmaier, 1959 and *S.calenssubcalens* Linsenmaier, 1959 (*nec* Mader 1933, an unavailable name) (Linsenmaier 1959, Linsenmaier 1997a, Linsenmaier 1997b). This latter taxon was later identified as *S.calenswesmaeli* Dahlbom, 1845 (Linsenmaier 1997a: 134, Linsenmaier 1997b: 287, Linsenmaier 1999: 254). However, Rosa and Vårdal (2015) discovered that the type of *Stilbumwesmaeli* is actually related to *S.cyanurum* (Forster, 1771) and the first available name for this taxon is *Stilbumwestermanni* Dahlbom, 1845. Rosa and Vårdal (2015) considered this to be a subspecies of *Stilbumcalens*, following Linsenmaier’s subspecific interpretation (Linsenmaier 1959, Linsenmaier 1997a, Linsenmaier 1997b).

**Figure 20.**
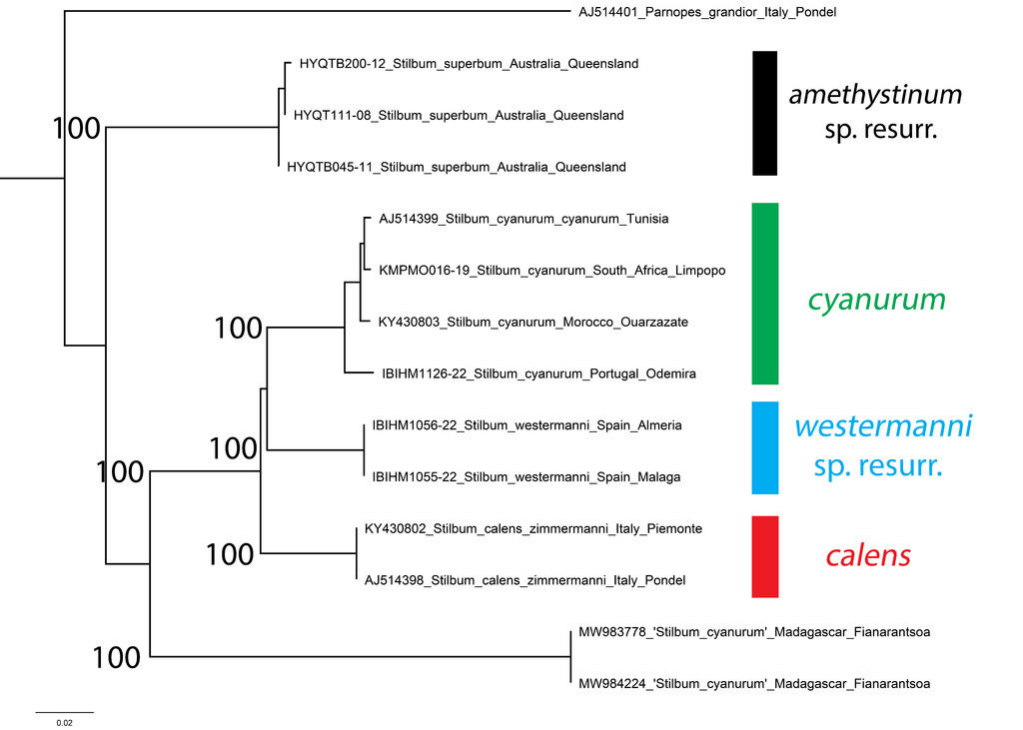
Phylogenetic tree (neighbour-joining) of *Stilbum* species, based on the DNA barcoding mitochondrial COI gene fragment. Numbers adjacent to branches represent bootstrap support (values of < 0.75 are omitted). The scale-bar indicates the % of sequence divergence.

**Figure 21.**
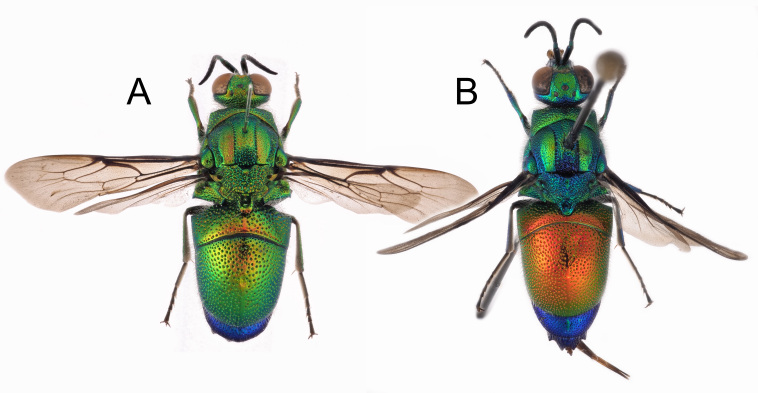
*Stilbum* species, dorsal view. **A** *Stilbumcyanurum* (Forster, 1771), male from Portugal, Odemira; **B** *Stilbumwestermanni* Dahlbom, 1845, female from Spain, Malaga.

Genetic results unambiguously support a species-level difference between *S.calenswestermanni* from Spain and Portugal and *S.calenszimmermanni* from Italy (Fig. 20). These taxa are separated by an average genetic distance of 6.62%, with bootstrap support of 100% for each clade. Both taxa are well-separated from *S.cyanurum* (Fig. 21A), by an average of 6.68% for *S.calenswestermanni* and by 6.86% for *S.calenszimmermanni*. Though collected over a large area from Portugal to South Africa, *S.cyanurum* shows low intraspecific variability, with average separation of 1.26% (range 0.47-1.89%). *Stilbumwestermanni* stat. nov. is, therefore, restored to species status (Fig. 21B). For now, we follow the interpretation of Linsenmaier that material from Central Europe should be referred to as *S.calenszimmermanni* until genetic samples are available from Siberia, the *locus typicus* of *S.calens* s. str.

The specimens DNA barcoded and identified in BOLD as *Stilbumcyanurum* from Madagascar (MW983778 and MW983223) are clearly distinct and actually belong to the species *Stilbumviride* Guérin-Méneville, 1842, the sole and endemic Madagascan *Stilbum* (Kimsey and Bohart 1991). Additionally, samples from Australia identified as ‘*S.superbum*’ are also clearly distinct. However, the name ‘*Stilbumsuperbum*’ is unavailable and it is likely an incorrect spelling of *Stilbumsplendidum* (Fabricius, 1775) that has recently been used on online sites. The Australian *Stilbum* species is clearly morphologically different from all other known species, but its taxonomic status has been confused. In literature, it has been commonly referred to as *S.splendidium* auct. or *S.amethystinum* auct. The type of the first taxon proved to be morphologically conspecific with *S.cyanurum* and considered to be a subjective synonym by Kimsey and Bohart (1991). The second was also considered to be a subjective synonym of *S.cyanurum* by Kimsey and Bohart (1991), but two syntypic specimens in London (Banks Collection, Natural History Museum) belong to a morphological separated species, characterised by smaller size and short malar spaces. Since this taxon is morphologically and genetically distinct, we here resurrect *S.amethystinum* (Fabricius, 1775) sp. resurr. from its previous synonymy with *S.cyanurum*. We also designate here the lectotype of *Chrysisamethystina* Fabricius, 1775 with one of the two specimens housed in the Banks Collection.

### Parnopes sp.

**Figure 22.**
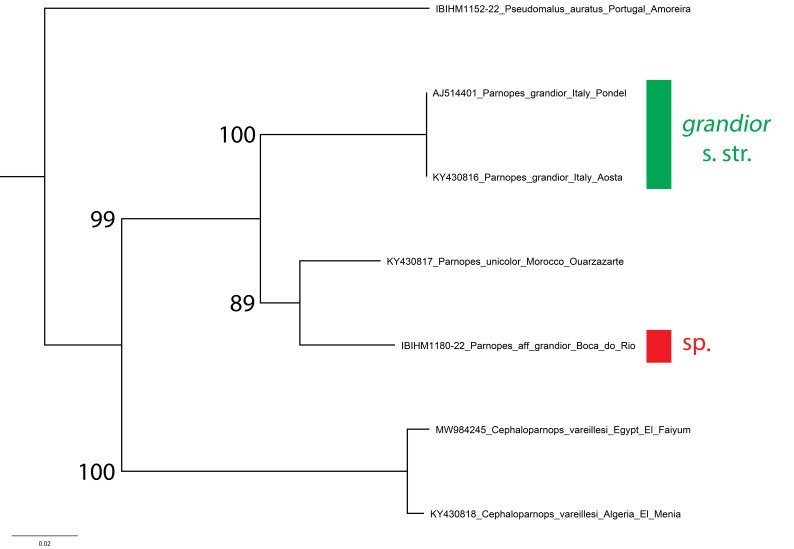
Phylogenetic tree (neighbour-joining) of *Parnopes* species, based on the DNA barcoding mitochondrial COI gene fragment. Numbers adjacent to branches represent bootstrap support (values of < 0.75 are omitted). The scale-bar indicates the % of sequence divergence.

The genetic sequence of this *Parnopes* specimen is strongly separated from the sequence of *P.grandior* from Italy by an average of 8.71% (Fig. 22). It is closer to a *P.unicolor* sequence from Morocco, but is still separated by 5.18%. Taken together, this taxon and *Parnopesunicolor* form a clade with bootstrap support of 89%, strongly separated from the *P.grandior* clade that has bootstrap support of 100%.

The discovery of another *Parnopes* species in the Iberian Peninsula is not so surprising as it seems, even though a name currently cannot be confidently assigned to this taxon. In recent years, a new species from Sardinia, *Parnopeslinsenmaieri* Agnoli, 1995 (described as subspecies of *Parnopesgrandior*) was described and another species was found through DNA barcoding Bulgarian specimens (BOLD, unpublished sequences). However, several new species of West Palearctic *Parnopes* will be described in an upcoming revision. These species have been overlooked because, classically, only three species were considered to be valid in the West Palearctic: *Parnopesgrandior* (known from Europe to central Asia), *P.unicolor* (northern Africa) and *P.glasunowi* (western Asia to central Asia) and specimens were identified, in part, based on the collecting locality and, in part, on body colouration. In this sense, all the variations and subspecies of *P.grandior* were considered to be only colour variation (Kimsey and Bohart 1991). The Portuguese female barcoded may be related to *Parnopesmarokkanus* Trautmann, 1927, a taxon not mentioned by Kimsey and Bohart (1991), Linsenmaier (1959), Linsenmaier (1968), Linsenmaier (1997b) and Linsenmaier (1999). In any case, many more genetic sequences and analyses are needed to understand the limits of variability within this genus. In fact, colouration is still seemingly very variable within populations, but could also represent the presence of valid sibling species.

### New additions to the Portuguese fauna

Thanks to this barcoding project, we analysed and added for the first time the following taxa to the list of the Portuguese species:

*Hedychridiumcaputaureum* Trautmann & Trautmann, 1919

*Hedychridiumcupritibiale* Linsenmaier, 1987

*Hedychridiumsevillanum* Linsenmaier, 1968

*Holopygafastuosa* Lucas, 1849

*Holopygajurinei* *sensu* Linsenmaier 1959

*Chrysiscastillana* du Buysson, 1894

*Chrysiscerastes* Abeille de Perrin, 1877

*Chrysisinsperata* Chevrier, 1870

*Chrysiscrossi* Rosa, sp. nov.

*Stilbumwestermanni* Dahlbom, 1845

## Supplementary Material

XML Treatment for Hedychridium calcarium

XML Treatment for Chrysis crossi

9999A089-92A7-580B-8D23-412D5C5F4013 10.3897/BDJ.11.e98743.suppl1 Supplementary material 1 IBI - Hymenoptera 02 Chrysididae library - Specimen details Data type Specimen data records Brief description The file includes information about all records in BOLD for the IBI - Hymenoptera 02 library. It contains collecting and identification data. The data are as downloaded from BOLD in the tsv format, without further processing. File: oo_776439.txt https://binary.pensoft.net/file/776439 Paolo Rosa, Thomas Wood, Sónia Ferreira

71CBE862-E86C-56DE-8A17-487A98C0CB7B 10.3897/BDJ.11.e98743.suppl2 Supplementary material 2 IBI - Hymenoptera 02 Chrysididae library - Specimen details Data type Specimen data records Brief description The file includes information about all records in BOLD for the IBI - Hymenoptera 02 library. It contains collecting and identification data. The data are as downloaded from BOLD in the DWC format, without further processing. File: oo_780452.txt https://binary.pensoft.net/file/780452 Paolo Rosa, Thomas Wood, Sónia Ferreira

C20B066D-4CF9-5955-BC33-29FD8ED6D033 10.3897/BDJ.11.e98743.suppl3 Supplementary material 3 IBI - Hymenoptera 02 Chrysididae library - DNA sequences Data type Genomic data, DNA sequences Brief description COI sequences in fasta format. Each sequence is identified by the BOLD ProcessID, species name, marker and GenBank accession number, separated by pipe. The data are as downloaded from BOLD. File: oo_776440.fas https://binary.pensoft.net/file/776440 Paolo Rosa, Thomas Wood, Sónia Ferreira

## Figures and Tables

**Table 1.** List of species that were collected and DNA barcoded within this project. # Indicates species with new BINs.

Genus	Species	IBI code	BOLD code	BOLD BIN	GenBank
*Chrysidea*	*Chrysideadisclusapumilionis* (Linsenmaier, 1987)#	INV12702	IBIHM1103-22	BOLD:AES3051	OP347205
*Chrysis*	*Chrysisandradei* Linsenmaier, 1959#	INV12728	IBIHM1129-22	BOLD:AES8383	OP347228
		INV12729	IBIHM1130-22		OP347302
	*Chrysisberlandi* Linsenmaier, 1959#	INV12682	IBIHM1083-22	BOLD:AES5679	OP347200
		INV12683	IBIHM1084-22		OP347265
		INV12734	IBIHM1135-22		OP347250
	*Chrysisblanchardi* Lucas, 1849#	INV12732	IBIHM1133-22	BOLD:AEU3313	OP347222
	*Chrysiscaeruliventris* Abeille de Perrin, 1878	INV12676	IBIHM1077-22	BOLD:AED3523	OP347173
	*Chrysiscastillana* Du Buysson, 1894	INV12731	IBIHM1132-22	BOLD:AED2289	OP347274
	*Chrysiscerastes* Abeille de Perrin, 1877#	INV12733	IBIHM1134-22	BOLD:AET4960	OP347293
	*Chrysisrutilans* Olivier, 1790#	INV12689	IBIHM1090-22	BOLD:AET4959	OP347268
	*Chrysischrysoprasina* Forster, 1853	INV12677	IBIHM1078-22	BOLD:AET4958	OP347283
	*Chrysischrysoscutella* Linsenmaier, 1959#	INV12743	IBIHM1144-22	BOLD:AES1459	OP347305
	*Chrysiscomparata* Lepeletier, 1806	INV12679	IBIHM1080-22	BOLD:AAU1528	OP347241
	*Chrysisconsanguinea* Mocsáry, 1889	INV12687	IBIHM1088-22	BOLD:AED0671	OP347212
	*Chrysiscortii* Linsenmaier, 1951	INV12670	IBIHM1071-22	BOLD:AAR9816	OP347211
	*Chrysiselegans* Lepeletier, 1806#	INV12673	IBIHM1074-22	BOLD:AES1460	OP347219
	*Chrysisemarginatula* Spinola, 1808	INV12672	IBIHM1073-22	BOLD:AED6786	OP347309
	*Chrysisfugax* Abeille de Perrin, 1878	INV12661	IBIHM1062-22	BOLD:AED3372	OP347230
	*Chrysisgermari* Wesmael, 1839#	INV12669	IBIHM1070-22	BOLD:AET6935	OP347229
	*Chrysisgracillimaaurofacies* (Trautmann, 1926)#	INV12663	IBIHM1064-22	BOLD:AES2863	OP347234
	*Chrysisgrohmanni* Dahlbom, 1854	INV12666	IBIHM1067-22	BOLD:AED6294	OP347210
	*Chrysishydropica* Abeille de Perrin, 1878#	INV12735	IBIHM1136-22	BOLD:AET2381	OP347243
	*Chrysisinsperata* Chevrier, 1870#	INV12774	IBIHM1175-22	BOLD:AET2383	OP347258
	*Chrysisintegra* Fabricius, 1787#	INV12741	IBIHM1142-22	BOLD:AET2382	OP347285
	*Chrysisirreperta* Linsenmaier, 1959#	INV12671	IBIHM1072-22	BOLD:AER8828	OP347244
	*Chrysislusitanica* Bischoff, 1910	INV12747	IBIHM1148-22	BOLD:ACQ6955	OP347197
	*Chrysismerceti* (Trautmann, 1926)#	INV12744	IBIHM1145-22	BOLD:AET3720	OP347260
	*Chrysismixta* Dahlbom, 1854#	INV12664	IBIHM1065-22	BOLD:AET3717	OP347207
	*Chrysismonticola* Linsenmaier, 1999#	INV12681	IBIHM1082-22	BOLD:AET3719	OP347289
		INV12730	IBIHM1131-22		OP347304
	*Chrysismysticalis* Linsenmaier, 1959#	INV12678	IBIHM1079-22	BOLD:AET3718	OP347245
		INV12736	IBIHM1137-22		OP347271
	*Chrysispeninsularis* du Buysson, 1887#	INV12667	IBIHM1068-22	BOLD:AES0122	OP347226
	*Chrysiscrossi* Rosa sp. nov.#	INV12727	IBIHM1128-22	BOLD:AES0121	OP347295
	*Chrysispulchella* Spinola, 1808	INV12665	IBIHM1066-22	BOLD:AED0619	OP347198
	*Chrysispulcherrima* Lepeletier, 1806#	INV12688	IBIHM1089-22	BOLD:AET0271	OP347172
	*Chrysispyrophana* Dahlbom, 1854#	INV12775	IBIHM1176-22	BOLD:AET0272	OP347213
	*Chrysisramburi* Dahlbom, 1854	INV12674	IBIHM1075-22	BOLD:AED5814	OP347263
	*Chrysissculpturata* Mocsáry, 1912	INV12684	IBIHM1085-22	BOLD:ABU6373	OP347310
	*Chrysisscutellarismarteni* Linsenmaier, 1951	INV12738	IBIHM1139-22	BOLD:ACM0910	OP347269
		INV12739	IBIHM1140-22		OP347185
	*Chrysissexdentata* Christ, 1791	INV12783	IBIHM1184-22	BOLD:ABU6376	OP347217
	*Chrysissplendidula* Rossi, 1790#	INV12740	IBIHM1141-22	BOLD:AES6413	OP347180
	*Chrysissubsinuata* Marquet, 1879#	INV12662	IBIHM1063-22	BOLD:AES4620	OP347214
	*Chrysisvaridens* Abeille de Perrin, 1878	INV12668	IBIHM1069-22	BOLD:AEE0312	OP347171
	*Chrysiszonata* Dahlbom, 1854#	INV12742	IBIHM1143-22	BOLD:AET8274	OP347199
*Chrysura*	*Chrysuraaustriaca* (Fabricius, 1804)	INV12690	IBIHM1091-22	BOLD:AAJ3472	OP347193
	*Chrysuracuprea* (Rossi, 1790)	INV12692	IBIHM1093-22	BOLD:AAP1055	OP347176
	*Chrysuradichroa* (Dahlbom, 1854)#	INV12696	IBIHM1097-22	BOLD:AET1511	OP347273
		INV12776	IBIHM1177-22		OP347215
	*Chrysurahybrida* (Lepeletier, 1806)	INV12726	IBIHM1127-22	BOLD:AAY6924	OP347177
	*Chrysurapurpureifrons* (Abeille de Perrin, 1878)#	INV12694	IBIHM1095-22	BOLD:AEU2029	OP347231
		INV12693	IBIHM1094-22	BOLD:AED1166	OP347286
		INV12695	IBIHM1096-22		OP347257
		INV12849	IBIHM1250-22		OP347296
		INV12850	IBIHM1251-22		OP347297
		INV12851	IBIHM1252-22		OP347221
	*Chrysuraradians* (Harris, 1776)	INV12697	IBIHM1098-22	BOLD:ABA8702	OP347225
	*Chrysurarefulgens* (Spinola, 1806)	INV12777	IBIHM1178-22	BOLD:ABA7395	OP347303
	*Chrysurarufiventris* (Dahlbom, 1854)	INV12699	IBIHM1100-22	BOLD:AEC6882	OP347290
	*Chrysurasimplex* (Dahlbom, 1854)	INV12691	IBIHM1092-22	BOLD:AAY6923	OP347189
	*Chrysurasulcata* (Dahlbom, 1845)	INV12700	IBIHM1101-22	BOLD:ABA7396	OP347249
	*Chrysuravaricornis* (Spinola, 1838)#	INV12698	IBIHM1099-22	BOLD:AET0222	OP347261
		INV12701	IBIHM1102-22		OP347192
*Hedychridium*	*Hedychridiumaereolum* du Buysson, 1892	INV12809	IBIHM1210-22	BOLD:AAY6930	OP347195
	*Hedychridiumanale* (Dahlbom, 1854)	INV12717	IBIHM1118-22	BOLD:AED4749	OP347270
	*Hedychridiumardens* (Coquebert, 1801)	INV12786	IBIHM1187-22	BOLD:AAK4640	OP347236
	*Hedychridiumbuyssoni* Abeille de Perrin, 1887#	INV12790	IBIHM1191-22	BOLD:AES9011	OP347196
	*Hedychridiumcaputaureum* Trautmann & Trautmann, 1919	INV12760	IBIHM1161-22	BOLD:AAU0775	OP347183
	*Hedychridiumvalesiense* Linsenmaier, 1959#	INV12804	IBIHM1205-22	BOLD:AET6828	OP347275
	*Hedychridiumchloropygum* du Buysson, 1888	INV12801	IBIHM1202-22	BOLD:AAE3258	OP347187
		INV12802	IBIHM1203-22		OP347240
	*Hedychridiumcupratum* (Dahlbom, 1854)	INV12806	IBIHM1207-22	BOLD:AAY6946	OP347255
		INV12807	IBIHM1208-22		OP347252
	*Hedychridiumcupritibiale* Linsenmaier, 1987#	INV12769	IBIHM1170-22	BOLD:AES9012	OP347239
	*Hedychridiumincrassatum* (Dahlbom, 1854)	INV12718	IBIHM1119-22	BOLD:AEE0029	OP347267
	*Hedychridiuminfans* Abeille de Perrin, 1878#	INV12768	IBIHM1169-22	BOLD:AES6837	OP347254
		INV12781	IBIHM1182-22		OP347191
	*Hedychridiumjucundum* Mocsáry, 1889#	INV12784	IBIHM1185-22	BOLD:AES6836	OP347262
		INV12785	IBIHM1186-22		OP347232
		INV12810	IBIHM1211-22	BOLD:AES6835	OP347170
		INV12811	IBIHM1212-22		OP347256
	*Hedychridiumkrajniki* Balthasar, 1946	INV12764	IBIHM1165-22	BOLD:AAZ0056	OP347237
	*Hedychridiummediocrum* Linsenmaier, 1987	INV12716	IBIHM1117-22	BOLD:AAE3260	OP347279
		INV12803	IBIHM1204-22		OP347308
	*Hedychridiummonochroum* du Buysson, 1888	INV12800	IBIHM1201-22	BOLD:AAY1978	OP347206
	*Hedychridiumreticulatum* Abeille de Perrin, 1878#	INV12763	IBIHM1164-22	BOLD:AER9655	OP347248
		INV12791	IBIHM1192-22		OP347203
		INV12792	IBIHM1193-22		OP347282
		INV12793	IBIHM1194-22		OP347301
	*Hedychridiumroseum* (Rossi, 1790)	INV12805	IBIHM1206-22	BOLD:AAE3259	OP347281
	*Hedychridiumsculpturatum* Abeille de Perrin, 1877#	INV12812	IBIHM1213-22	BOLD:AET6828	OP347181
		INV12813	IBIHM1214-22	BOLD:AAE3258	OP347276
	*Hedychridiumscutellare* (Tournier, 1878)#	INV12761	IBIHM1162-22	BOLD:AES0428	OP347291
		INV12788	IBIHM1189-22		OP347175
	*Hedychridiumsevillanum* Linsenmaier, 1968#	INV12765	IBIHM1166-22	BOLD:AET6827	OP347242
		INV12767	IBIHM1168-22	BOLD:AES0429	OP347259
		INV12789	IBIHM1190-22		OP347223
	*Hedychridiumcalcarium* Rosa sp. nov.#	INV12794	IBIHM1195-22	BOLD:AET9698	OP347204
		INV12795	IBIHM1196-22		OP347307
		INV12796	IBIHM1197-22		OP347169
	*Hedychridiumsubroseumprochloropygum* Linsenmaier, 1959#	INV12715	IBIHM1116-22	BOLD:AES2437	OP347209
	*Hedychridiumvachali* Mercet, 1915#	INV12766	IBIHM1167-22	BOLD:AET6826	OP347280
*Hedychrum*	*Hedychrumlongicolle* Abeille de Perrin, 1877	INV12723	IBIHM1124-22	BOLD:AED0972	OP347298
	*Hedychrummicanseuropaeum* Linsenmaier, 1959	INV12724	IBIHM1125-22	BOLD:AAK4644	OP347194
	*Hedychrumniemelai* Linsenmaier, 1959	INV12721	IBIHM1122-22	BOLD:AAU1294	OP347300
	*Hedychrumnobile* (Scopoli, 1763)	INV12722	IBIHM1123-22	BOLD:AAK4644	OP347266
		INV12771	IBIHM1172-22		OP347168
	*Hedychrumrutilans* Dahlbom, 1854	INV12720	IBIHM1121-22	BOLD:AAM3491	OP347220
	*Hedychrumviridiaureum* Tournier, 1877	INV12719	IBIHM1120-22	BOLD:AAM3491	OP347294
		INV12770	IBIHM1171-22	BOLD:AAK4643	OP347247
		INV12782	IBIHM1183-22	BOLD:AAM3491	OP347174
*Holopyga*	*Holopygacalida* Linsenmaier, 1951#	INV12705	IBIHM1106-22		OP347202
		INV12754	IBIHM1155-22	BOLD:AES0090	OP347227
	*Holopygalucida* (Lepeletier, 1806)#	INV12708	IBIHM1109-22	BOLD:AES0091	OP347182
	*Holopygafastuosa* (Lucas, 1849)	INV12709	IBIHM1110-22	BOLD:AAZ6194	OP347292
		INV12710	IBIHM1111-22		OP347190
		INV12780	IBIHM1181-22		OP347253
		INV12753	IBIHM1154-22	BOLD:AAY6928	OP347216
	*Holopygafervida* (Fabricius, 1781)	INV12706	IBIHM1107-22	BOLD:ACV6331	OP347208
		INV12757	IBIHM1158-22	BOLD:AAY9735	OP347287
	*Holopygagenerosa* (Förster, 1853)	INV12712	IBIHM1113-22	BOLD:AAZ6194	OP347288
		INV12713	IBIHM1114-22		OP347201
	*Holopygainflammata* (Förster, 1853)#	INV12759	IBIHM1160-22	BOLD:AET1451	OP347284
	*Holopygajurinei* Chevrier, 1862#	INV12714	IBIHM1115-22		OP347233
		INV12756	IBIHM1157-22	BOLD:AET1450	OP347235
	*Holopygasimilis* Mocsáry, 1889	INV12787	IBIHM1188-22	BOLD:AED0274	OP347178
	*Holopygamerceti* Kimsey, 1991#	INV12755	IBIHM1156-22	BOLD:AES1216	OP347186
*Parnopes*	*Parnopes* sp.#	INV12779	IBIHM1180-22	BOLD:AET2814	OP347218
*Philoctetes*	*Philoctetesabeillei* du Buysson, 1892#	INV12704	IBIHM1105-22	BOLD:AEU5026	OP347179
	*Philoctetespunctulatus* (Dahlbom, 1854)#	INV12703	IBIHM1104-22	BOLD:AEU5027	OP347264
		INV12749	IBIHM1150-22		OP347272
		INV12750	IBIHM1151-22		OP347238
*Pseudochrysis*	*Pseudochrysishumboldti* (Dahlbom, 1845)#	INV12659	IBIHM1060-22	BOLD:AEU3425	OP347246
	*Pseudochrysisincrassata* (Spinola, 1838)#	INV12660	IBIHM1061-22	BOLD:AEU3426	OP347278
	*Pseudomalusauratus* (Linnaeus, 1758)	INV12751	IBIHM1152-22	BOLD:AAH8217	OP347251
	*Pseudomalusviolaceus* (Scopoli, 1763)	INV12752	IBIHM1153-22	BOLD:ABX9998	OP347299
*Spintharina*	*Spintharinacuprata* (Dahlbom, 1854)#	INV12657	IBIHM1058-22	BOLD:AET6497	OP347277
	*Spintharinaversicolor* (Spinola, 1808)	INV12656	IBIHM1057-22	BOLD:AAJ3630	OP347224
*Stilbum*	*Stilbumwestermanni* Dahlbom, 1845#	INV12654	IBIHM1055-22	BOLD:AES3895	OP347311
		INV12655	IBIHM1056-22		OP347306
	*Stilbumcyanurum* (Forster, 1771)	INV12725	IBIHM1126-22	BOLD:AAJ4180	OP347184
*Trichrysis*	*Trichrysiscyanea* (Linnaeus, 1758)	INV12658	IBIHM1059-22	BOLD:AAH7935	OP347188
